# Toward Interpretable Voice-Based Parkinson’s Disease Screening via Joint Transfer Function–Feature–Classifier–Ensemble Selection

**DOI:** 10.3390/bioengineering13091037

**Published:** 2026-09-06

**Authors:** Xiaolei Yuan, Xinyue Zhang, Hui Xu, Qing Ye, Canxing Yuan, Hui Li

**Affiliations:** 1 Department of Neurology, Unit 1, Longhua Hospital, Shanghai University of Traditional Chinese Medicine, No. 725 Wanping South Road, Xuhui District, Shanghai 200032, China; yuanxiaolei@shutcm.edu.cn (X.Y.); xyzhang@shutcm.edu.cn (X.Z.); lh2749@shutcm.edu.cn (Q.Y.); 2 Shanghai Minhang District Traditional Chinese Medicine Hospital, Shanghai 201101, China; xhiuhux@163.com

**Keywords:** Parkinson’s disease, voice biomarkers, feature selection, competitive swarm optimizer, joint optimization

## Abstract

Parkinson’s disease (PD) diagnosis relies on subjective clinical examination of motor signs that can be mild, intermittent, or absent early in the disease course, motivating objective, low-cost, non-invasive markers for earlier, more consistent detection. Voice recordings, acquirable with nothing more than a microphone, are a strong candidate, and this study develops a machine learning pipeline for voice-based PD screening built on the Competitive Swarm Optimizer (CSO), which jointly searches the acoustic feature subset, classifier configuration, and binarization transfer function, instead of optimizing the feature subset alone as most prior pipelines do. Evaluated on two public, subject-grouped voice datasets, Oxford and Naranjo, against five baselines under an identical protocol across 20 runs per method, our proposed pipeline attains the highest mean balanced accuracy on Naranjo with 0.847 and the second-highest on Oxford with 0.810, accuracies consistent with the wider voice-based PD screening literature; because this evidence comes from two small, single-recording-protocol, retrospective public datasets of 31 and 80 subjects each, we present it as an initial, encouraging step toward a first-pass triage or between-visit monitoring tool, pending external validation on a prospectively collected, multi-site cohort, not as a standalone diagnostic instrument. As an initial step toward clinical interpretability, we check which acoustic features are selected most consistently across 20 repeated runs of the proposed pipeline against established physiological correlates of Parkinsonian dysphonia; agreement between any two runs’ complete feature subsets is weak, but pitch period entropy, a nonlinear-dynamical measure of aperiodic pitch period variability, is selected far more often than chance on both datasets, consistent with the underlying pathophysiology and not merely predictive. These results support voice-based, metaheuristic-optimized screening as a plausible, interpretable, low-burden tool for telemedicine and home monitoring.

## 1. Introduction

Parkinson’s disease (PD) is the second most common neurodegenerative disorder worldwide, arising from progressive degeneration of dopaminergic neurons in the substantia nigra [1]. Its global burden has risen sharply alongside population aging: an estimated 3.15 million individuals were living with PD in 1990, a number that grew to 11.8 million by 2021 [2] and is projected to reach 25.2 million by 2050 [3]. Diagnosis remains primarily clinical, relying on the observation of cardinal motor signs, namely bradykinesia, rigidity, resting tremor, and postural instability, under established frameworks such as the Movement Disorder Society (MDS) Clinical Diagnostic Criteria [4], typically supported by assessment of the patient’s response to levodopa therapy. This process is inherently subjective and is particularly prone to misdiagnosis at early disease stages, when motor signs may be mild, intermittent, or absent [5]. Given that the optimal timing and long-term benefit of dopaminergic and prospective disease-modifying therapies remain active questions under clinical investigation [6], and that any such therapy can only be offered once PD is recognized in the first place, there exists a pressing clinical need for objective, low-cost, and non-invasive markers capable of enabling earlier and more consistent detection than motor examination alone.

Among candidate biomarkers, vocal impairment has attracted particular attention. Hypokinetic dysarthria is estimated to affect the large majority of individuals with PD [7,8], arising from rigidity and reduced range of motion in the laryngeal, respiratory, and articulatory musculature. These physiological changes produce measurable perturbations in the acoustic signal, namely increased cycle-to-cycle variation in fundamental frequency (jitter) and a reduced harmonics-to-noise ratio (HNR) reflecting incomplete glottal closure [9], increased cycle-to-cycle variation in amplitude (shimmer), and altered nonlinear measures of vocal-fold vibration such as recurrence period density entropy and detrended fluctuation analysis [10]. Such vocal changes can emerge early in the disease course, at times preceding or co-occurring with the earliest overt motor signs by as much as a decade [8], and, unlike gait or tremor assessment, can be captured remotely, repeatedly, and inexpensively using nothing more than a standard microphone. This renders voice analysis a particularly attractive candidate for scalable, low-burden screening, including telemedicine and home-monitoring scenarios that cannot readily accommodate in-person motor examination.

Motivated by this, a substantial and rapidly growing body of work has applied machine learning to acoustic features extracted from sustained-phonation or connected-speech recordings for automated PD detection [11]. Given that the number of extractable acoustic measures is often large relative to the number of available recordings, feature selection is commonly employed to reduce dimensionality, limit overfitting, and improve interpretability. Wrapper approaches built around population-based metaheuristic algorithms have become an increasingly popular choice for this task, owing to their derivative-free search mechanisms and their capacity to explore high-dimensional, non-convex feature spaces without requiring restrictive assumptions on the underlying data distribution.

Despite this progress, several limitations persist across the existing literature. First, the large majority of studies treat the choice of classifier as fixed and predetermined, optimizing only the feature subset; the complementary question of which classifier, or combination of classifiers, best exploits a given feature subset is rarely optimized jointly within the same search process. Even recent work that does optimize feature selection and a classifier together typically keeps the classifier type itself fixed and tunes only its hyperparameters jointly with the feature subset [12], instead of searching over which classifier to use in the first place. This separation implicitly assumes that an optimal feature subset is invariant to the classifier that will ultimately consume it, an assumption that is not generally supported, since different classifiers exploit different aspects of the feature space and may therefore favor markedly different feature subsets. Second, feature selection and classifier selection are commonly performed sequentially, not jointly, such that suboptimal decisions made at the feature-selection stage cannot be corrected during classifier selection, and vice versa; this two-stage pipeline risks converging to a solution that is locally optimal for each subproblem but jointly suboptimal overall, a pattern still evident in very recent PD-specific pipelines that pair a filter-based feature-selection stage with a separately optimized classification ensemble [13]. Third, and less widely recognized, the transfer function that converts a metaheuristic’s continuous search space into the binary feature masks it actually evaluates is itself treated as a fixed, externally chosen design decision, not something the search can determine: the S-shaped and V-shaped families remain the default convention across the literature, yet comparative evaluations spanning multiple transfer functions on the same task consistently find that no single choice dominates across datasets and algorithms [14], such that this too is a design choice usually settled by ad hoc pre-selection or trial and error instead of by the optimization itself, alongside the feature subset and classifier it jointly searches over. Taken together, these limitations motivate a unified search framework capable of jointly optimizing feature subsets, classifier configurations, and the transfer function governing binarization, one that is validated across multiple, independent datasets to ensure robustness and generalizability of the resulting diagnostic model.

Therefore, this study proposes a joint transfer function-, feature-, and classifier-optimization pipeline for voice-based PD screening, built on the plain Competitive Swarm Optimizer (CSO). The main contributions of this research are as follows:

- We propose a novel paradigm for joint search over the acoustic feature subset, the classifier that exploits it, and the transfer function used to binarize the search itself, within a single optimization encoding, instead of optimizing feature selection alone or fixing a binarization scheme in advance.
- We extend this joint encoding with a per-particle transfer-function-selector segment, so a plain Competitive Swarm Optimizer searches over which of five candidate binarization schemes governs each particle, instead of every particle in the population sharing one transfer function fixed before the search begins.
- We compare the proposed method against five recently published metaheuristic feature-selection approaches applied specifically to PD datasets, under one shared encoding, fitness function, and evaluation protocol, with statistical significance testing, thereby demonstrating its competitiveness.
- We conduct a sensitivity analysis of CSO’s social-term coefficient φ for this specific joint optimization task, empirically confirming that the value consistent with the original algorithm’s own reported parameter settings at this study’s problem dimensionality is also the best-performing one, instead of assuming that value without checking it.
- We check the acoustic features selected by the proposed pipeline’s best-performing run on each dataset against established physiological correlates of Parkinsonian dysphonia, as an initial, illustrative step toward assessing the biological plausibility and potential clinical interpretability of the resulting screening model.

The remainder of this paper is organized as follows. Section 2 reviews related work on PD diagnosis, voice biomarkers, and metaheuristic feature selection. Section 3 describes the datasets employed and the proposed methodology. Section 4 presents the experimental results, including comparative performance evaluation and statistical significance testing. Section 5 discusses the clinical implications and limitations of the proposed approach. Section 6 concludes the paper.

## 2. Related Work

### 2.1. Current Diagnostic Practice and the Case for Objective Biomarkers

Clinical diagnosis of PD remains anchored to the MDS Clinical Diagnostic Criteria [4]: a judgment, made by a trained examiner, of whether bradykinesia is present together with rest tremor and/or rigidity, refined by supportive features and exclusion criteria and typically corroborated by the patient’s response to levodopa therapy. Because this judgment rests on the visual and manual assessment of motor signs that can be subtle, intermittent, or altogether absent early in the disease course, diagnostic accuracy is known to vary considerably with examiner experience and disease duration, and misdiagnosis remains common enough at early stages to be a recurring concern in the movement-disorders literature [5]. This has motivated a broader search for objective biomarkers that do not depend on a clinician’s subjective interpretation of motor signs. Dopaminergic imaging (DAT-SPECT, PET), cerebrospinal-fluid and blood-based α-synuclein assays, genetic testing, and, more recently, wearable and other digital-sensing modalities [15] have all been investigated as candidate objective markers; each can support diagnosis or staging, but each also carries some combination of high cost, invasiveness, specialized equipment, or limited accessibility outside tertiary care centers. Voice and speech recordings, by contrast, can be acquired with nothing more than a standard microphone, at negligible marginal cost, and without any physical contact with the patient, a combination of properties that renders them an unusually attractive candidate among the emerging digital-biomarker modalities, and the specific focus of this study.

### 2.2. Acoustic and Speech Biomarkers of Parkinsonian Dysphonia

Hypokinetic dysarthria, characterized by reduced vocal loudness, monopitch, imprecise articulation, and breathy or hoarse voice quality, is estimated to affect the large majority of individuals with PD at some point in the disease course [7,8]. Its physiological basis lies in rigidity and bradykinesia affecting the laryngeal, respiratory, and articulatory musculature: reduced vocal-fold adduction force and control lead to incomplete or irregular glottal closure, reduced respiratory support lowers subglottal pressure and vocal intensity, and reduced range of motion in the articulators blurs consonant and vowel targets [9]. These physiological changes are reflected in several well-established acoustic correlates, namely increased cycle-to-cycle perturbation in fundamental frequency (jitter) and amplitude (shimmer), reduced harmonics-to-noise ratio (HNR) reflecting incomplete glottal closure, reduced vowel space area reflecting imprecise articulation, and altered nonlinear dynamical measures of vocal-fold vibration, including recurrence period density entropy (RPDE), detrended fluctuation analysis (DFA), and pitch period entropy (PPE) [10], that are more sensitive than classical perturbation measures to the aperiodic, weakly chaotic component of Parkinsonian phonation [9]. Given that these physiological changes can begin before overt motor signs are clinically apparent, acoustic and broader speech biomarkers have increasingly been proposed as tools for prodromal detection and longitudinal monitoring, not merely post-diagnosis screening [8,16]. This physiological grounding is what would make the specific acoustic features a wrapper-based pipeline selects clinically meaningful, not merely predictive; Section 5.2 returns to this point, checking the selected features of the proposed pipeline’s best-performing run against it.

### 2.3. Metaheuristic Feature Selection for PD Detection: Progress and Methodological Gaps

Wrapper-based feature selection couples a search procedure with a downstream classifier’s cross-validated performance as the objective to optimize. Population-based metaheuristics, being derivative-free and requiring no assumption of a smooth or convex fitness landscape, have become a standard choice of search procedure for this task across biomedical applications generally [17]. Within voice-based PD detection specifically, a fast-growing line of work applies swarm- and evolution-inspired algorithms to select acoustic feature subsets ahead of a downstream classifier, most commonly encoding the search space as a binary mask over the candidate feature set: particle-swarm-, butterfly-, and whale-inspired variants [18], particle swarm optimization jointly tuning the feature subset and classifier hyperparameters [19], a modified grey wolf optimizer [20], a modified hunger games search [21], a quantum mayfly optimizer [22], a genetic algorithm with filter-based pre-selection [23], a beluga whale optimizer [24], and hybrids chaining two algorithms in sequence, namely a whale-to-grey-wolf cascade [25] and a chaotic grey wolf–dragonfly cascade [26].

As discussed in Section 1, the classifier consuming the selected features is fixed and predetermined in the large majority of this work, rarely optimized jointly with the feature subset within a single search process. Even where classifier hyperparameters are tuned alongside the feature subset [19], the classifier type itself is still chosen in advance, not searched over. This omission is not merely a matter of scope: because different classifiers weight, combine, and interact with input features in fundamentally different ways, the feature subset best suited to one classifier need not be the one best suited to another, so fixing the classifier a priori risks discarding feature subsets that would perform poorly under the assumed classifier but well under an alternative one that was never considered. Extending the search to include the classifier itself, however, is not a trivial enlargement of the existing formulations; it substantially increases the dimensionality and, more importantly, the combinatorial complexity of the search space, since feature and classifier choices interact instead of contributing independently to fitness, which is precisely the setting in which premature convergence and loss of population diversity become most damaging to search quality. This is the gap that the encoding proposed in this study is designed to close, by searching a higher-dimensional, joint feature-and-classifier space without sacrificing search quality relative to feature-selection-only formulations.

## 3. Materials and Methods

### 3.1. Datasets and Study Population

Two public, retrospective, previously collected acoustic datasets are used, which are presented as follows.

**Oxford Parkinson’s Disease Voice Dataset** (https://archive.ics.uci.edu/dataset/174/parkinsons (accessed on 9 August 2026)) [10] comprises 195 sustained phonation, vowel /a/, recordings from 31 subjects, 23 Parkinsonian and 8 healthy control, of whom 147 recordings, 75.4%, are labeled Parkinsonian and 48, 24.6%, healthy control, that is, class-imbalanced at the recording level. Each recording is described by 22 acoustic features, including fundamental-frequency perturbation measures, jitter variants; amplitude perturbation measures, shimmer variants; harmonics-to-noise ratio; and nonlinear dynamical measures, namely RPDE, DFA, spread1/2, D2, and PPE. Subjects contribute multiple recordings each, approximately six on average, which is the reason subject-grouped cross-validation is required instead of a plain record-level split.

**Naranjo Replicated Acoustic Features Dataset** (https://archive.ics.uci.edu/dataset/489/parkinson+dataset+with+replicated+acoustic+features (accessed on 9 August 2026)) [27] comprises 240 recordings from 80 subjects, 40 Parkinsonian and 40 healthy controls, perfectly class-balanced at the subject level, each subject contributing exactly 3 repeated recordings. Each recording is described by 45 acoustic features: jitter, shimmer, and amplitude-perturbation variants, together with a gender covariate, extracted specifically to support test–retest reliability analysis across repeated recordings of the same speaker. This renders subject-grouped evaluation especially important for this dataset, since its very design guarantees near-identical repeated measurements per subject.

### 3.2. Data Preprocessing and Subject-Grouped Cross-Validation

No missing values are present in either dataset; features are used in their raw scale, with per-fold standardization applied inside each classifier’s own pipeline, not globally, so as to avoid leaking validation-fold statistics into training. Every fitness evaluation performed by every optimizer in this study uses 5-fold cross-validation, grouped by subject identifier and stratified by class label. This guarantees that all recordings from a given subject fall entirely within a single fold, such that no classifier ever trains on one recording from a subject while being validated on another recording from that same subject. Out-of-fold predictions from all 5 folds are concatenated before any performance metric is computed, such that every reported score is computed over the entire dataset under a strict train/validation separation, not averaged across fold-level scores.

### 3.3. Transfer Function-, Feature-, and Classifier-Joint Optimization Encoding

Every optimizer in this study searches over a shared, particle-based encoding: a continuous position vector of length 5+D+5, where the first 5 is the number of transfer functions, *D* is the number of acoustic features in the dataset, 22 for Oxford and 45 for Naranjo, and the final 5 is the number of potential classifiers. The first 5 dimensions are decoded as a one-hot scheme, that is, only one transfer function with the highest value in the corresponding dimension is selected. The first *D* dimensions are binarized, via each optimizer’s own transfer function, into a feature-selection mask over the *D* acoustic features. The last 5 dimensions correspond to five candidate base classifiers, each with recent precedent as the wrapped classifier in its own evolutionary or metaheuristic feature-selection study: support vector machine (SVM), an RBF-kernel classifier calibrated via 5-fold Platt scaling for probability estimates [28] and paired with PSO-based feature selection for Alzheimer’s disease diagnosis in [29]; random forest (RF) with 100 trees, paired with an improved genetic algorithm for feature selection in [30]; extreme gradient boosting (XGBoost) with 100 trees, paired with a multiobjective evolutionary algorithm for biomarker feature selection in [31]; *k*-nearest neighbors (KNN) with k=5, paired with physics-inspired metaheuristic feature selection in [32]; and logistic regression (LogReg), paired with particle swarm optimization for feature selection in chronic kidney disease diagnosis in [33]. These five are chosen to span linear, instance-based, and both bagged and boosted tree-ensemble decision boundaries, so that the joint search can in principle favor whichever inductive bias best matches each dataset’s feature space.

The last 5 dimensions are decoded the same way for every algorithm in this study: independent on/off switches, such that every classifier whose switch is on trains on the same selected feature subset, and the ensemble’s out-of-fold prediction is the unweighted average of the active classifiers’ predicted probabilities, i.e., equal-weight soft voting. Optimizing which base learners join a voting ensemble of classifiers, instead of fixing the ensemble’s membership in advance, has precedent in [34], albeit via hard majority voting over discrete class labels instead of the probability-averaging scheme adopted here. Applying this multi-classifier ensemble decoding uniformly, instead of letting each baseline fall back to whatever single-classifier scheme its own binarization naturally produces, keeps the comparison in Section 4 isolated to each method’s search strategy: every algorithm is optimizing over, and evaluated on, the identical solution space, so that a performance difference cannot be attributed to one method being handed a weaker or stronger classifier-selection scheme than another. In addition, if a particle ever decodes to an empty feature mask or an empty classifier mask, one bit is uniformly at random forced to be active to keep the candidate solution evaluable.

**Figure 1:**
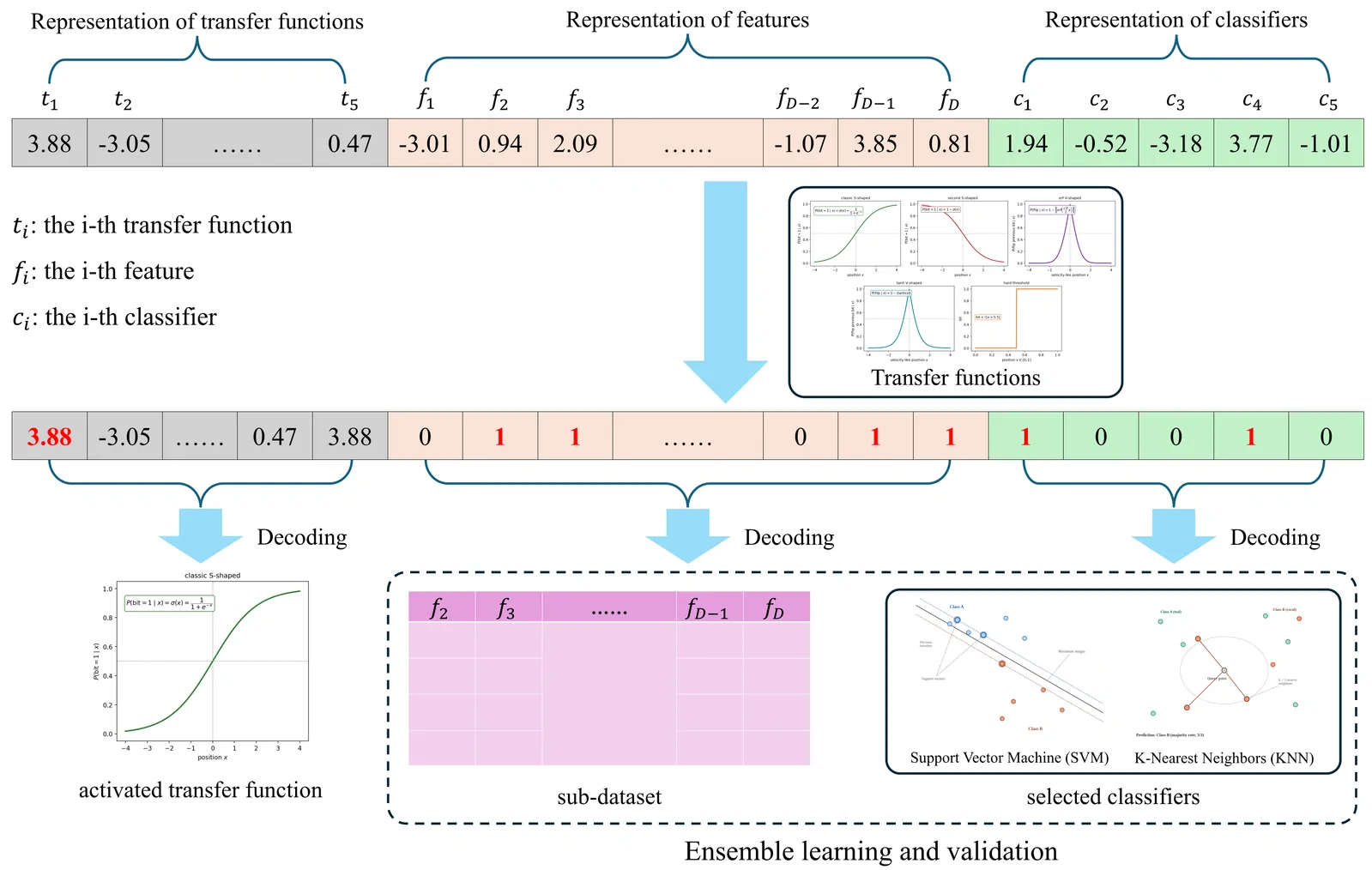
Worked example of the encoding and decoding pipeline. A continuous particle position of length 5+D+5 splits into 5 transfer function scores ($t_{1},\ldots ,t_{5}$), *D* feature scores ($f_{1},\ldots ,f_{D}$), and 5 classifier scores ($c_{1},\ldots ,c_{5}$). Each segment passes through a transfer function to produce a binary string; thresholded-off features/classifiers (0) are excluded, and the remainder (1, highlighted) are decoded into a feature mask, here selecting the sub-dataset restricted to columns $f_{2},f_{3},\ldots ,f_{D-1},f_{D}$, and a classifier mask, here selecting SVM and KNN. Every classifier in the mask trains on the same feature subset and is combined by equal-weight soft voting before 5-fold subject-grouped cross-validation.

Figure 1 demonstrates this working pipeline end-to-end.

### 3.4. Transfer Functions for Binarization

The swarm- and population-based metaheuristics used in this study were, in their original formulation, designed for continuous optimization: a particle’s position is a real-valued vector, and the search dynamics, attraction toward better solutions, and so on, are defined in the continuous space. Joint feature-and-classifier selection is inherently discrete, since each dimension of a solution must resolve to a binary decision; that is, a given feature or classifier is either selected or not. A transfer function bridges this mismatch: it maps each continuous position value to a probability or a fixed threshold that determines the corresponding bit, so that an optimizer’s continuous-space search dynamics can be reused unchanged while the solution actually evaluated is binary.

**Figure 2:**
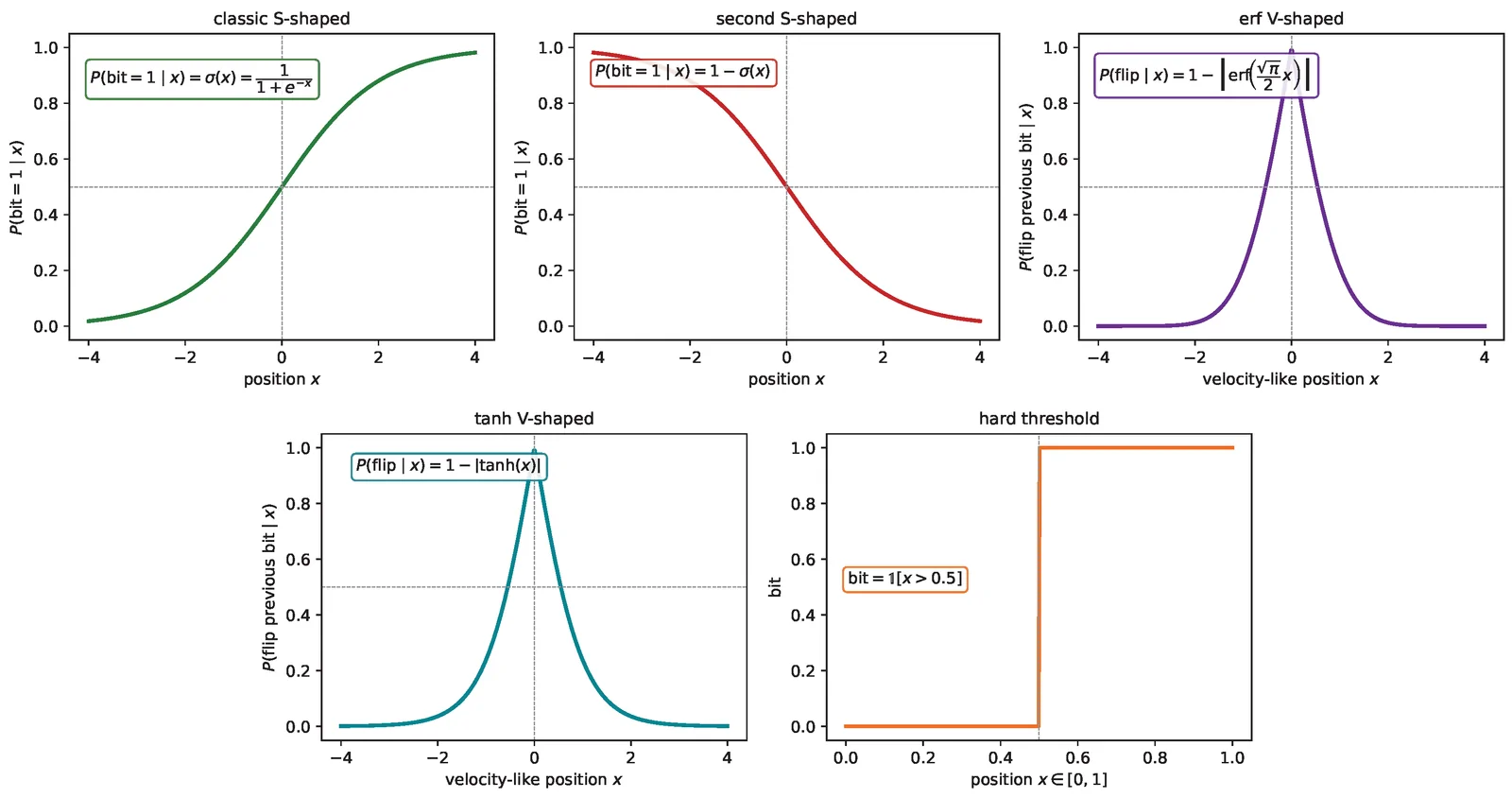
The five transfer functions used in this study: Top row, left to right: classic S-shaped (Equation (1)) [14]; second S-shaped (Equation (2)) [18]; erf V-shaped (Equation (3)) [18,35]. Bottom row, centered under the top row: tanh V-shaped (Equation (4) [35]; hard threshold (Equation (5)) [21], used natively in [0,1].

Five such transfer functions are adopted in this research, shown in Figure 2.

The classic stochastic S-shaped transfer function adopts the logistic sigmoid to give the probability that a bit is set, as formulated in Equation (1).

(1) $$\sigma \left(x\right)=\frac{1}{1+e^{-x}},\;\mathrm{Pr}[\mathrm{bit}=1]=\sigma \left(x\right),$$

so that selection probability increases monotonically with *x*; this remains an active choice of binarization scheme in recent metaheuristic feature-selection studies [14].

A second S-shaped variant, following Hashemi et al. [18], reuses the same logistic sigmoid but decodes in the opposite direction from Equation (1), which is defined in Equation (2).

(2) Pr[bit=1]=1−σ(x),

Another V-shaped curve, originally defined by Mirjalili and Lewis [35] and adopted within the hybrid transfer-function scheme of [18], instead governs whether the previous bit flips, with flip probability in Equation (3).

(3) $$\mathrm{Pr}\left[\mathrm{bit}\;\mathrm{flips}\right]=1-V\left(x\right),\;V\left(x\right)=\left|\mathrm{erf}\left(\frac{\sqrt{\pi }}{2}x\right)\right|,$$

which peaks at x=0 (where V(0)=0) and decays toward 0 as |x| grows (where V(x)→1).

Additionally, a tanh V-shaped curve, following Mirjalili and Lewis’s [35] own V-shaped family, uses the hyperbolic tangent in place of the error function:

(4) $$\mathrm{Pr}\left[\mathrm{bit}\;\mathrm{flips}\right]=1-V_{2}\left(x\right),\;V_{2}\left(x\right)=\left|\tanh\left(x\right)\right|,$$

with the same flip-the-previous-bit mechanic and the same peaks-at-zero, decays-as-|x|-grows shape as Equation (3), merely a cheaper closed form. Finally, a hard-threshold binarization [21] applies directly to positions that live natively in [0,1], with no stochastic component:

(5) $$\mathrm{bit}=\left\{\begin{array}{cc} 1 & x>0.5,\\ 0 & \mathrm{otherwise}. \end{array}\right.$$

### 3.5. Fitness Function

Every candidate solution is scored by a single scalar fitness, combining diagnostic performance with a feature-parsimony penalty in Equation (6):

(6) $$\mathrm{fitness}=\omega \cdot \left(1-\mathrm{BalAcc}\right)+\left(1-\omega \right)\cdot \frac{\left|S\right|}{D}$$

where BalAcc is the out-of-fold balanced accuracy, the average of sensitivity and specificity and robust to Oxford’s class imbalance [36], achieved by the decoded feature subset and ensemble of classifiers, |S| is the number of selected features, *D* is the total number of available features, and ω=0.9 weights diagnostic performance nine times as heavily as feature count, consistent with the common practice in the bio-inspired PD feature-selection literature, e.g., [37], of weighting diagnostic accuracy substantially more heavily than parsimony. This value ensures a modest reduction in feature count is rewarded only when it does not come at a material cost to balanced accuracy. Additionally, lower fitness is better, and every optimizer in this study is implemented as a minimizer.

### 3.6. Our Proposed Pipeline: The Joint Optimization via Competitive Swarm Optimizer

The contribution of this study is not a new metaheuristic operator but a new paradigm for handling metaheuristic-based feature selection efficiently. As reviewed in Section 2.3, published metaheuristic pipelines for PD voice screening almost universally treat the transfer function, the feature subset, and the classifier configuration as three separate decisions, of which only the feature subset is determined via metaheuristic search, while the other two components are configured by hand according to expert judgment. This section describes the pipeline that instead searches all three jointly, within the single 5+D+5-dimensional encoding of Section 3.3 and the fitness of Section 3.5, using the Competitive Swarm Optimizer (CSO) [38] as the search engine that explores this space generation by generation.

#### 3.6.1. Basic CSO

The Competitive Swarm Optimizer (CSO) was originally proposed by Cheng and Jin in 2015 [38], and departs from the attractor-based update rule common to PSO, where every particle is pulled toward the personal best and global best solution. CSO instead structures each generation as a set of pairwise competitions: only the loser of each pair updates and learns from the winner particle and the centroid position. This design is intended to preserve swarm diversity and allocate the computational budgets to the promising solutions. Concretely, in each generation, the even-numbered population of *N* particles is randomly paired into N/2 one-on-one competitions. Within each pair, the fitter particle, denoted as $v_{w}^{t}$, passes to the next generation unchanged, while the weaker particle, denoted as $v_{l}^{t}$, updates its velocity and position toward the winner and the centroid of the swarm, as described in Equation (7):

(7) $$\begin{array}{cc} & v_{l}^{t+1}=R_{1}\cdot v_{l}^{t}+R_{2}\cdot \left(x_{w}^{t}-x_{l}^{t}\right)+\varphi \cdot R_{3}\cdot \left({\bar{x}}^{t}-x_{l}^{t}\right)\\ & x_{l}^{t+1}=x_{l}^{t}+v_{l}^{t+1} \end{array}$$

where $x_{w}^{t}$ and $x_{l}^{t}$ are the winner’s and loser’s positions, respectively, $\bar{x}$ is the centroid of the swarm, $R_{1}$, $R_{2}$, and $R_{3}$ are independent uniform random vectors, and ϕ is CSO’s social-term control coefficient, where the sensitivity analysis for ϕ in this specific joint optimization task is provided in Section 4.3.

#### 3.6.2. The Implementation of Our Proposed Framework

**Figure 3:**
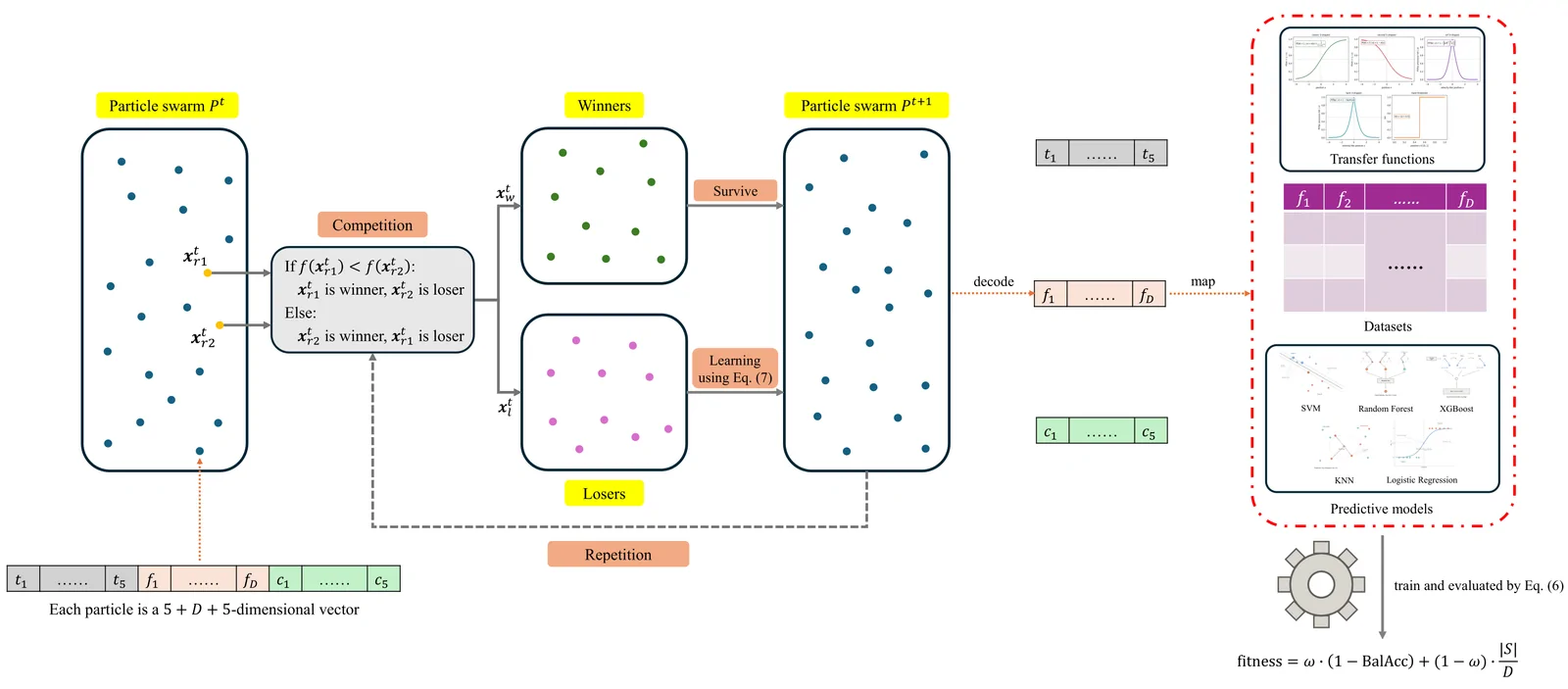
One full generation of the proposed pipeline.

Figure 3 traces one full generation of the proposed pipeline end-to-end, tying together the encoding of Section 3.3, the transfer-function candidates of Section 3.4, the CSO update of Section 3.6.1, and the fitness of Section 3.5 into the single procedure that this study actually runs.

Each particle in the swarm $P^{t}$ is a 5+D+5-dimensional vector $\left[t_{1},\ldots ,t_{5},f_{1},\ldots ,f_{D},c_{1},\ldots ,c_{5}\right]$. Every generation, particles are randomly paired and compared based on the competition mechanism in CSO; each pair’s winner survives unchanged into $P^{t+1}$, while its loser’s entire position is updated by Equation (7) before being decoded and re-evaluated. Decoding a position for the updated particle individual consists of three steps:

1. Transfer-function selection: The leading segment $\left[t_{1},\ldots ,t_{5}\right]$ is argmaxed to pick one of the 5 candidate transfer functions without a binarization step.
2. Feature and classifier mapping: The selected candidate binarizes the trailing $\left[f_{1},\ldots ,f_{D},c_{1},\ldots ,c_{5}\right]$ segment based on the specific transfer function. The resulting *D*-bit feature mask selects which of the dataset’s *D* acoustic-feature columns are retained, as mapped in Figure 3; the resulting 5-bit classifier mask selects which of the five candidate classifiers are included in that particle’s ensemble.
3. Training and evaluation: The selected classifiers are trained on the selected feature subset under the subject-grouped cross-validation protocol of Section 3.2 and combined into an equal-weight soft-voting ensemble; the resulting out-of-fold balanced accuracy and feature ratio are combined into the scalar fitness computed by Equation (6).

The resulting fitness value is what determines that particle’s winner/loser status the next time it is paired, closing the loop shown at the bottom of Figure 3: decode, map, train, and evaluate happen every time a loser updates, generation after generation, for every particle in the swarm.

### 3.7. Comparison Methods

Five metaheuristic feature-selection methods from recently published papers, specifically focusing on PD diagnosis, are re-implemented to enable a controlled, like-for-like comparison against the proposed framework: binary grey wolf optimizer (BGWO) [39] and a whale-optimization-then-grey-wolf refinement cascade (HybridGWO) [25], mayfly algorithm with a quantum-rotation-gate mutation operator (QMFO) [22], and binary particle swarm optimization (BPSO) as well as butterfly optimization algorithm (BBOA) with a hybrid S-shaped or V-shaped transfer function [18]. Table 1 summarizes the detailed parameter settings of these algorithms.

Every optimizer is compared under an equal fitness-evaluation budget, $FE_{max}$, instead of an equal generation count, since different algorithms consume different numbers of fitness evaluations per generation: the hybrid S-shaped/V-shaped transfer function used by BPSO/BBOA, for example, evaluates two binarization candidates per individual per generation. A population size of 30 and a fitness-evaluation budget of $FE_{max}=3000$ per run are used throughout. Each algorithm–dataset combination is run with multiple independent seeds, seed =2026+50×i, where *i* is the run index in {0,1,…,19}, i.e., 20 independent, non-overlapping seeds per algorithm–dataset combination. This fair and detailed comparison against the five baselines, carried out across multiple environments, uses enough independent runs to support the statistical testing described in Section 4.

### 3.8. Evaluation Metrics

For every run, the out-of-fold predictions used to compute fitness are also used to report a full panel of standard diagnostic-performance metrics, computed once per run from the same confusion matrix, whose entries are true positives (TP), true negatives (TN), false positives (FP), and false negatives (FN), with the Parkinsonian class taken as positive:

(8) $$\mathrm{Accuracy}=\frac{TP+TN}{TP+TN+FP+FN}$$

(9) $$\mathrm{Sensitivity}\;\left(\mathrm{Recall}\right)=\frac{TP}{TP+FN}$$

(10) $$\mathrm{Specificity}=\frac{TN}{TN+FP}$$

(11) $$\mathrm{BalAcc}=\frac{\mathrm{Sensitivity}+\mathrm{Specificity}}{2}$$

(12) $$\mathrm{Precision}=\frac{TP}{TP+FP}$$

(13) $$F1=2\cdot \frac{\mathrm{Precision}\cdot \mathrm{Sensitivity}}{\mathrm{Precision}+\mathrm{Sensitivity}}$$

(14) $$\mathrm{ROC-AUC}=\mathrm{Pr}\left(\hat{p}\left(x^{+}\right)>\hat{p}\left(x^{-}\right)\right)$$

where $\hat{p}\left(x\right)$ is the predicted probability of the Parkinsonian class for a recording *x*, and $x^{+}$, $x^{-}$ are independently drawn Parkinsonian and healthy-control recordings, respectively. That is, ROC-AUC, formulated in Equation (14), is the probability that the model ranks a randomly drawn positive case above a randomly drawn negative one, equivalently the area under the curve traced by the true positive rate versus the false positive rate as the decision threshold varies, unlike Equations (8)–(13), which are all evaluated at the single decision threshold of 0.5 used elsewhere in this study. Sensitivity and specificity are reported alongside the more ML-conventional accuracy/F1/AUC metrics because they are the standard vocabulary for evaluating a clinical screening tool: sensitivity in Equation (9) quantifies the fraction of true PD cases the pipeline would flag, since missed cases carry a direct clinical cost, while specificity in Equation (10) quantifies the fraction of healthy individuals correctly spared unnecessary follow-up. BalAcc in Equation (11) is the same quantity used inside the fitness function, so the primary search objective and the headline reported metric are one and the same, not two different notions of accuracy. Feature-selection ratio (|S|/D) and the active classifier(s) are also recorded for every run; the identity of the specific features selected is not logged for every run in the current experiment pipeline, only recovered separately for a single best-performing run per dataset, as Section 5.2 discusses.

## 4. Results

This section compares the proposed CSO pipeline against the five baselines from Section 3.7 on both datasets, under the equal fitness-evaluation budget and multi-seed protocol described.

### 4.1. Comparison on the Oxford Dataset

Table 2 summarizes the mean ± standard deviation of every reported metric on the Oxford dataset across all independent runs of each algorithm.

**Figure 4:**
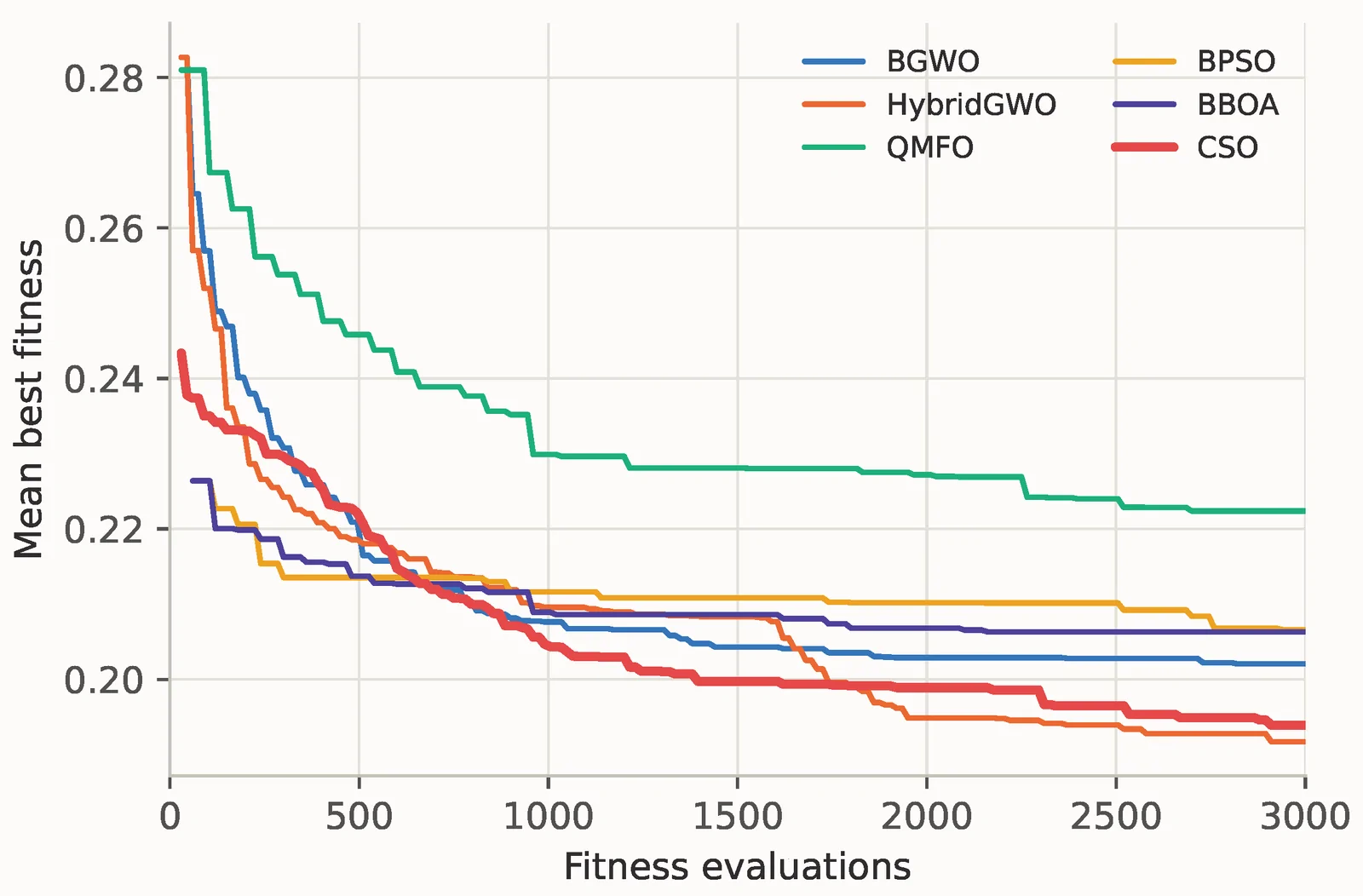
Mean best-fitness convergence on the Oxford dataset. Lower is better; CSO is highlighted.

Figure 4 shows the mean best-fitness trajectory, as computed in Equation (6), against the number of fitness evaluations consumed, averaged over all independent runs of each algorithm.

**Figure 5:**
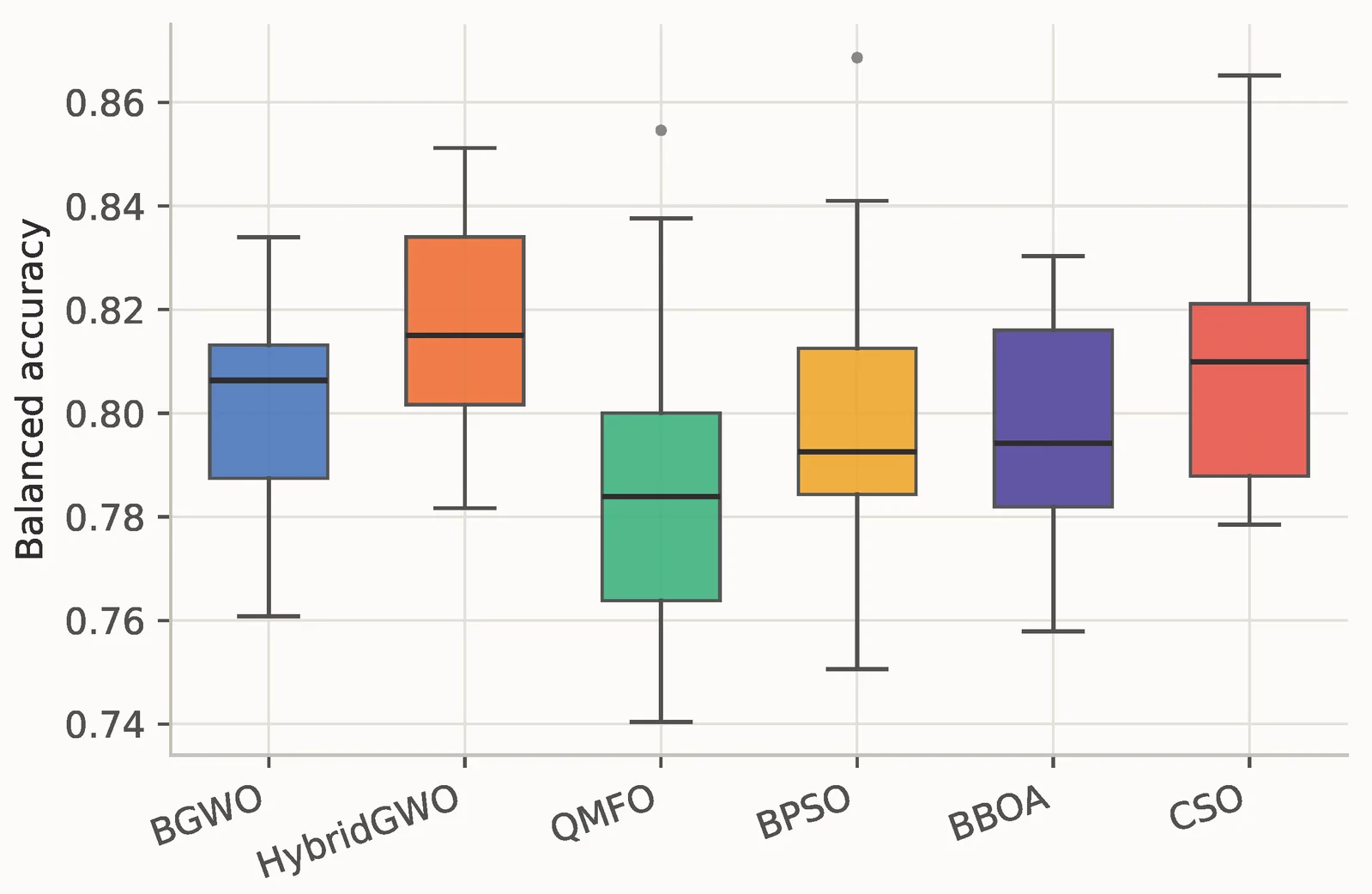
Balanced-accuracy distribution across runs on the Oxford dataset. Dots denote outlier runs falling outside this range.

Figure 5 shows the distribution of out-of-fold balanced accuracy, as computed in Equation (11), achieved by each algorithm across its independent runs.

**Figure 6:**
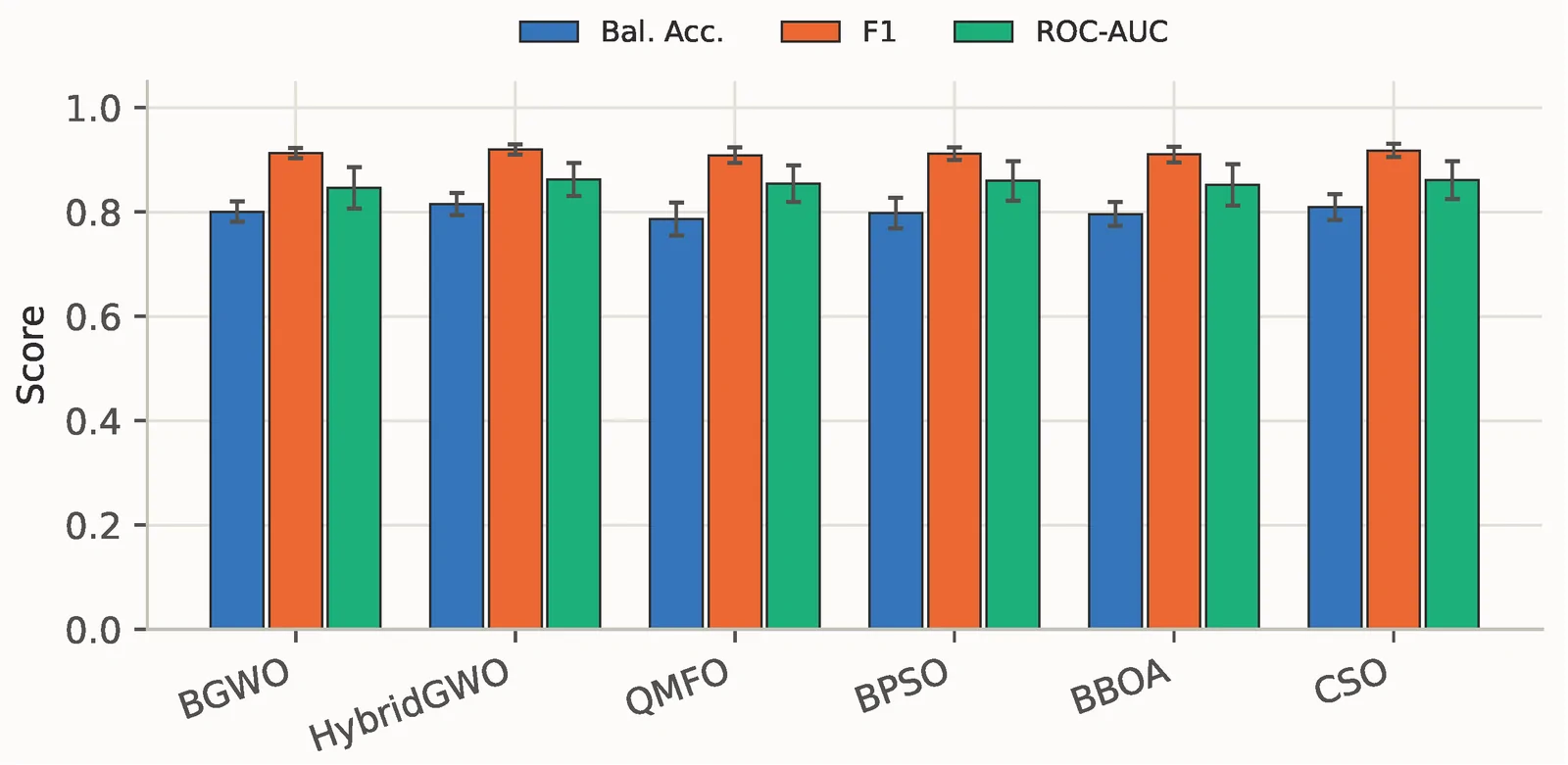
Mean ± standard deviation of balanced accuracy, F1, and ROC-AUC on the Oxford dataset.

Figure 6 reports the mean and standard deviation of balanced accuracy, F1, as computed in Equation (13), and ROC-AUC, as computed in Equation (14), for each algorithm.

**Figure 7:**
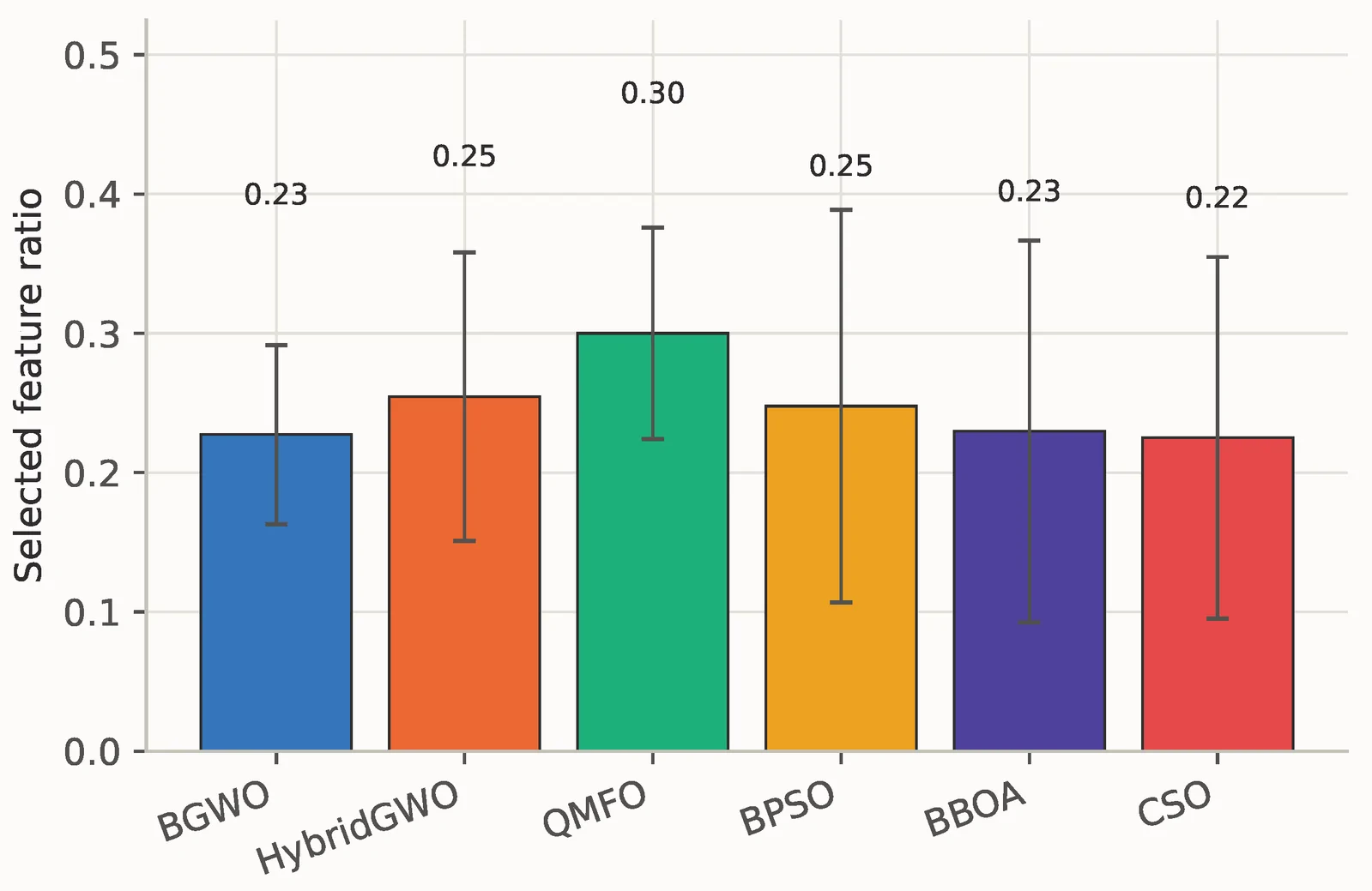
Mean ± standard deviation of the selected feature ratio on the Oxford dataset.

Figure 7 reports the mean and standard deviation of the selected feature ratio, |S|/D in Equation (6), for each algorithm.

### 4.2. Comparison on the Naranjo Dataset

Table 3 summarizes the mean ± standard deviation of every reported metric on the Naranjo dataset across all independent runs of each algorithm.

**Figure 8:**
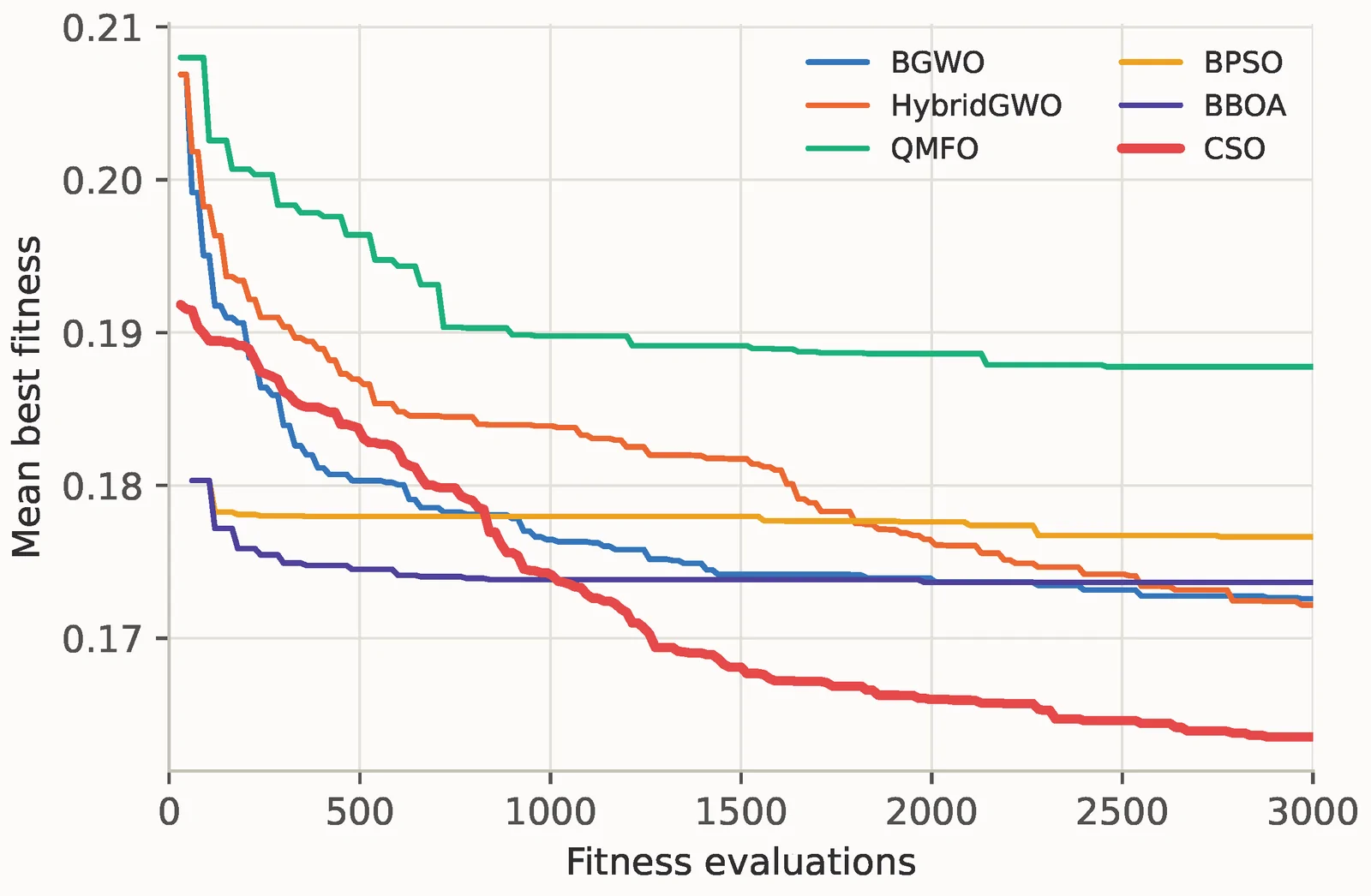
Mean best-fitness convergence on the Naranjo dataset. Lower is better; CSO is highlighted.

Figure 8 shows the mean best-fitness trajectory, as computed in Equation (6), against the number of fitness evaluations consumed, averaged over all independent runs of each algorithm.

**Figure 9:**
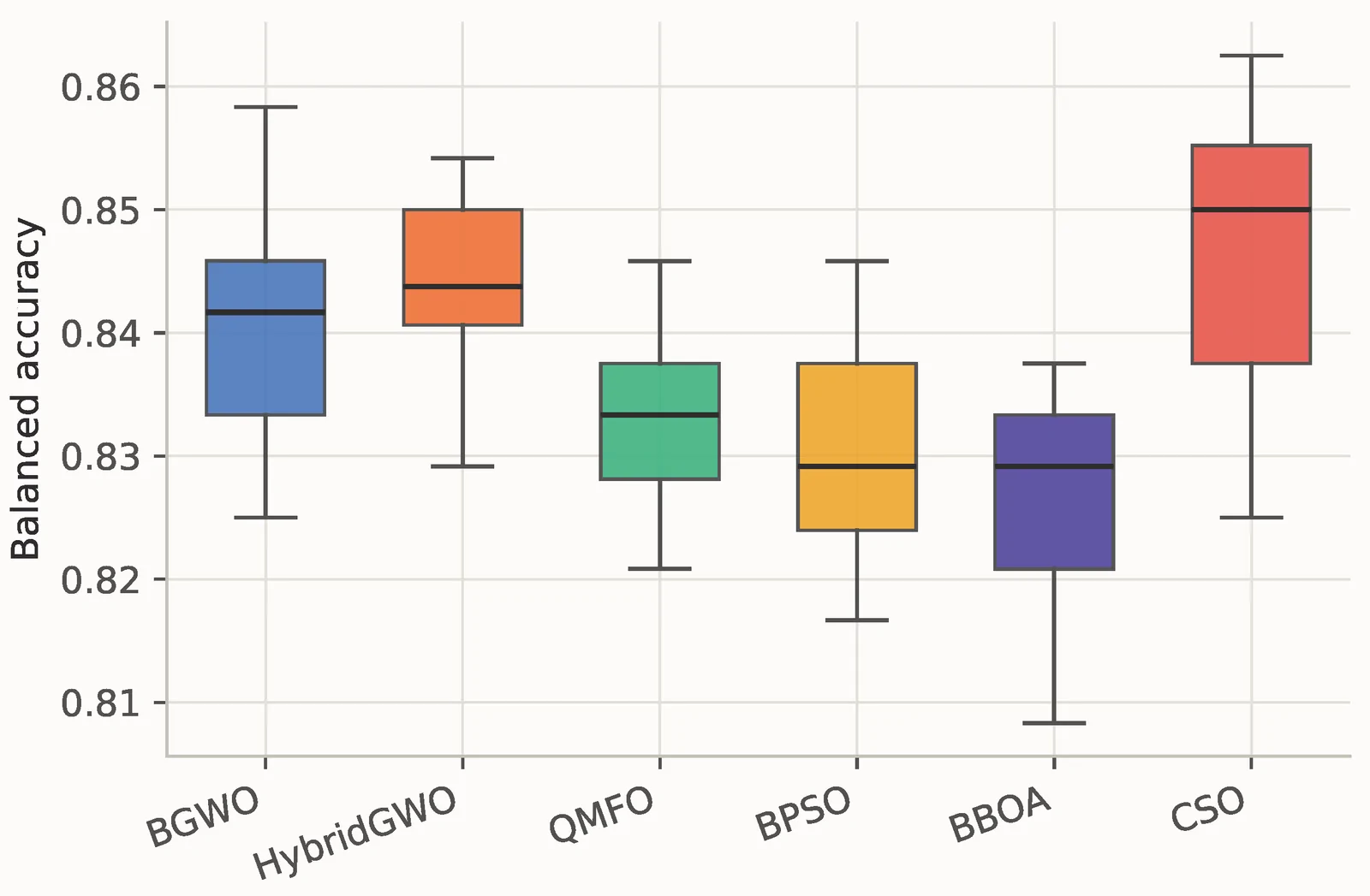
Balanced-accuracy distribution across runs on the Naranjo dataset.

Figure 9 shows the distribution of out-of-fold balanced accuracy, as computed in Equation (11), achieved by each algorithm across its independent runs.

**Figure 10:**
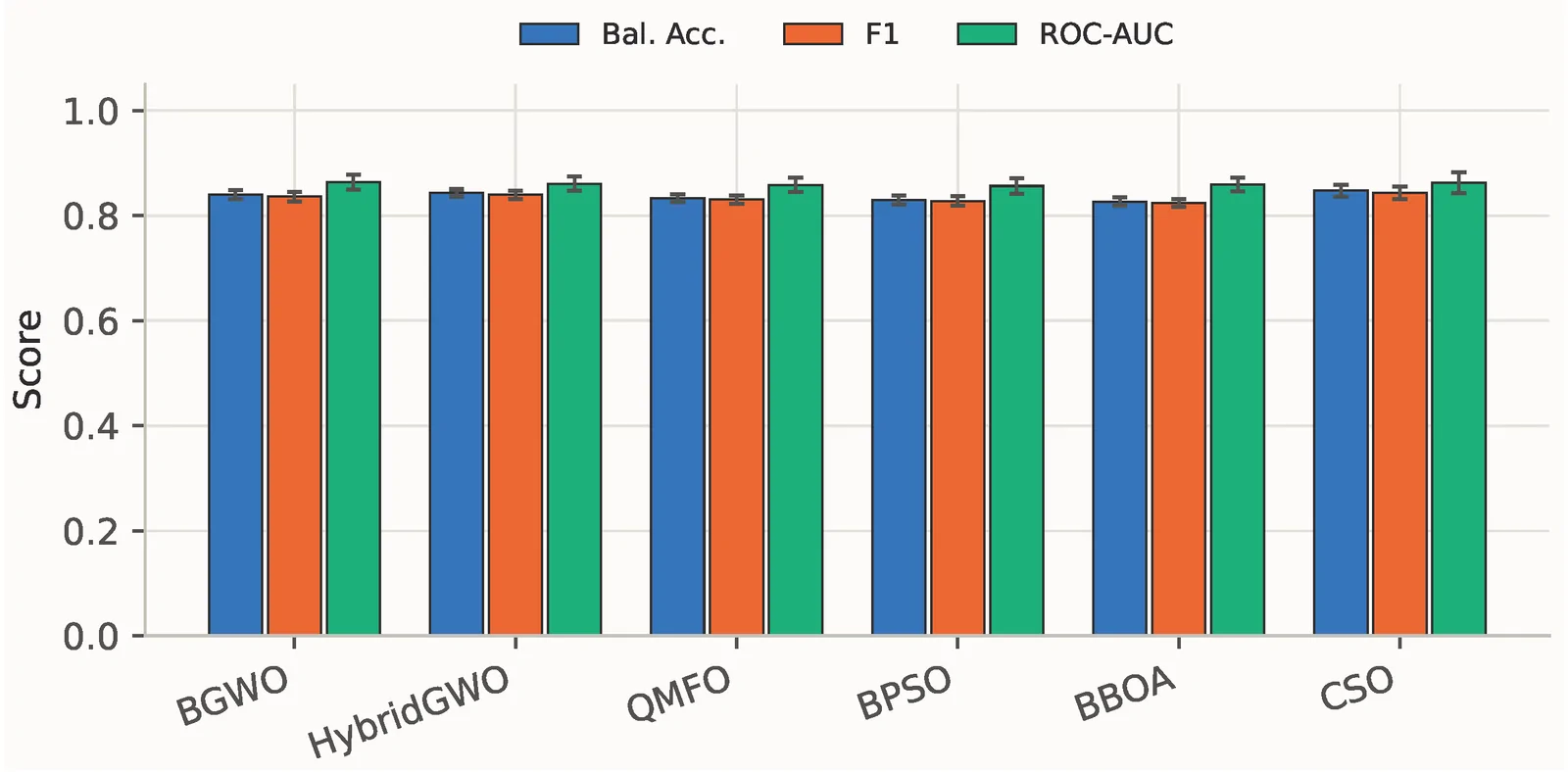
Mean ± standard deviation of balanced accuracy, F1, and ROC-AUC on the Naranjo dataset.

Figure 10 reports the mean and standard deviation of balanced accuracy, F1, as computed in Equation (13), and ROC-AUC, as computed in Equation (14), for each algorithm.

**Figure 11:**
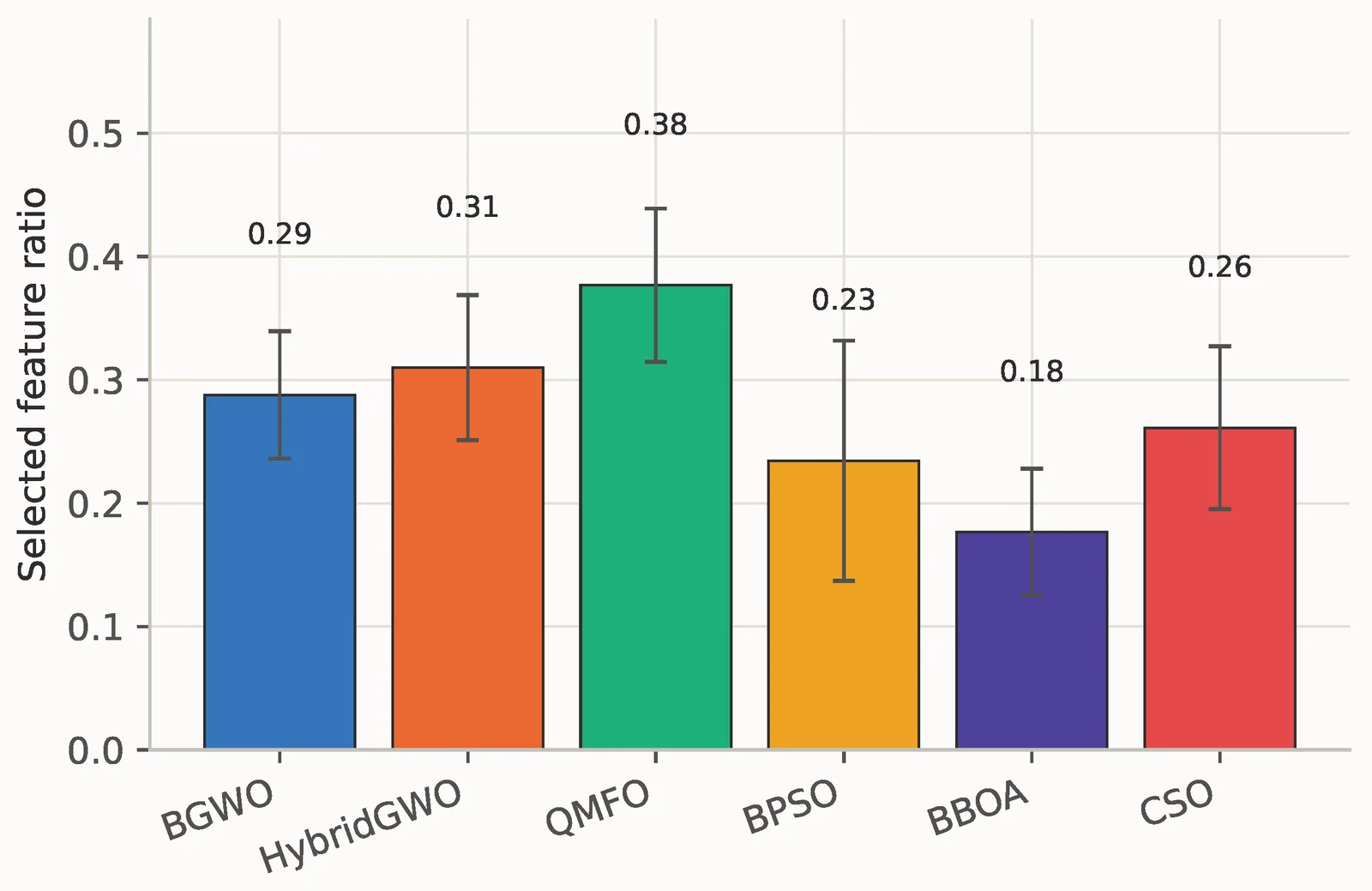
Mean ± standard deviation of the selected feature ratio on the Naranjo dataset.

Figure 11 reports the mean and standard deviation of the selected feature ratio, |S|/D in Equation (6), for each algorithm.

### 4.3. Sensitivity Analysis of the CSO Social-Term Coefficient φ

As noted in Table 1, the proposed pipeline uses φ=0 throughout the comparison of Section 4.1 and Section 4.2, consistent with Cheng and Jin’s own reported parameter settings [38], which pair φ=0 with problem dimensionality of 100 or fewer, a condition this study’s 5+D+5 joint encoding satisfies. To check the sensitivity of the hyperparameter φ in this research, φ is swept over {0.00,0.05,0.10,…,0.50}, overriding this default value with each fixed candidate in turn while every other setting is held fixed: population size 30, $FE_{max}=3000$, and 20 independent seeds per φ value per dataset.

Table 4 and Table 5 summarize the mean ± standard deviation of best fitness, balanced accuracy, and selected feature ratio attained under each φ, on Oxford and Naranjo respectively.

**Figure 12:**
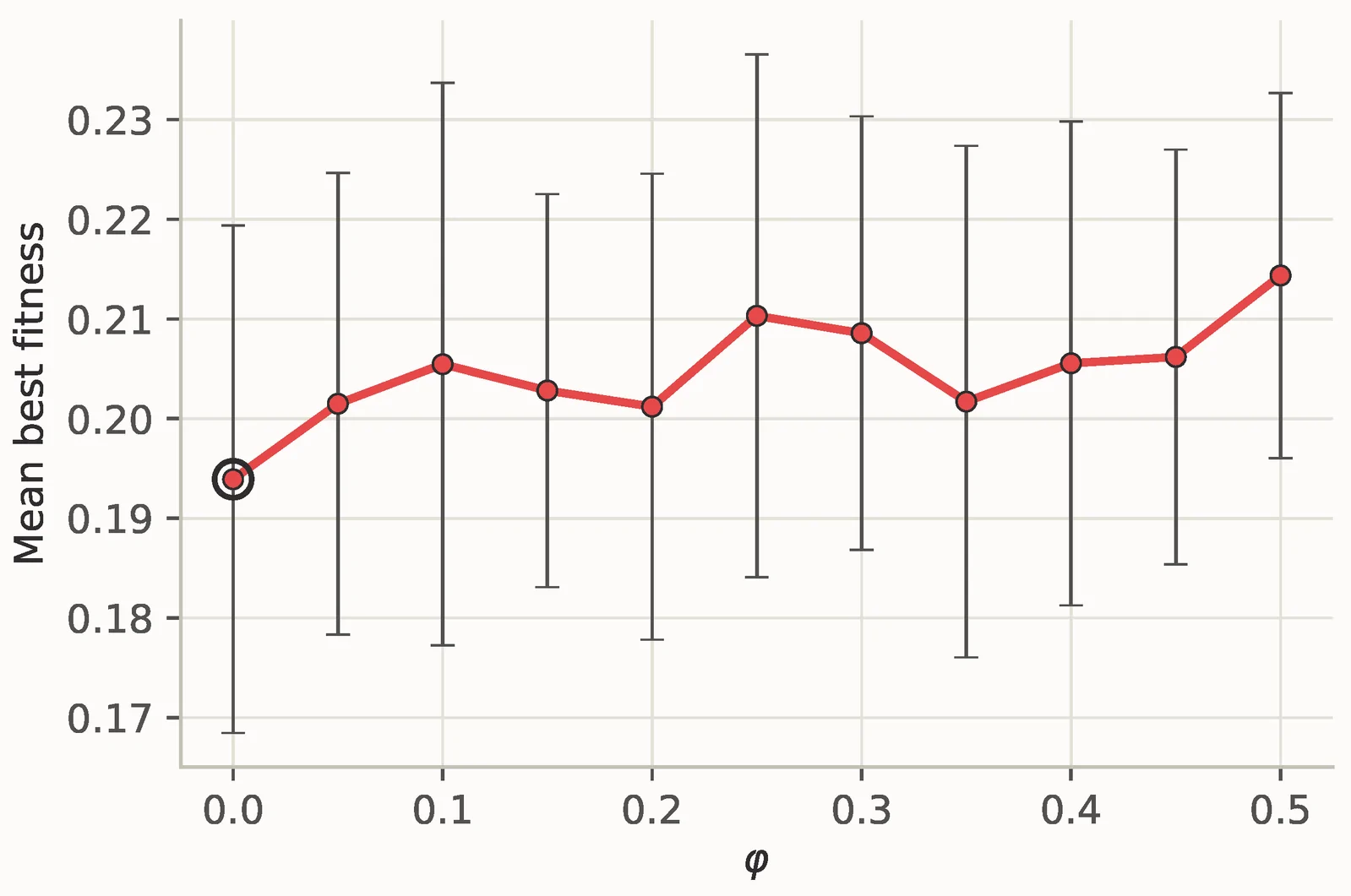
Mean ± standard deviation of best fitness against φ on the Oxford dataset. The circled marker is the best-performing φ.

**Figure 13:**
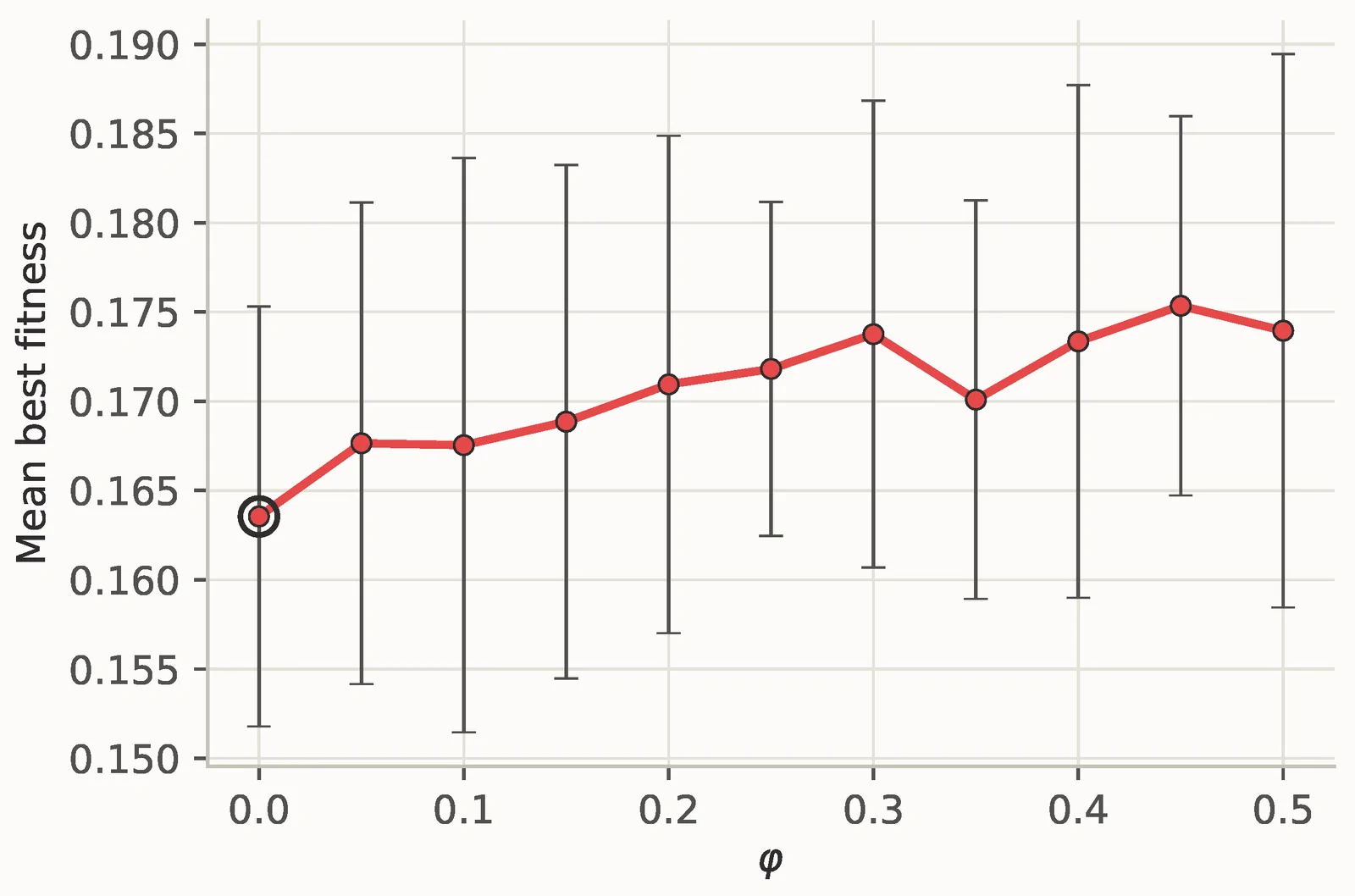
Mean ± standard deviation of best fitness against φ on the Naranjo dataset. The circled marker is the best-performing φ.

Figure 12 and Figure 13 show the mean ± standard deviation of the best fitness (Equation (6)) attained under each φ, on Oxford and Naranjo respectively; the circled marker highlights the best-performing value. On both datasets, φ=0 attains the lowest mean best fitness of the eleven values swept.

Table 4 and Table 5 above, instead of a separate figure, decompose this into the balanced-accuracy and feature-ratio terms of the fitness function. φ=0 attains the highest mean balanced accuracy on both datasets, and on Naranjo, the decline as φ increases is close to monotonic. The feature ratio, unlike best fitness and balanced accuracy, shows no clear trend with φ on either dataset: Oxford’s minimum falls at φ=0.05 and Naranjo’s at φ=0, consistent with the fitness function of Equation (6) weighting feature ratio an order of magnitude less than accuracy, ω=0.9.

Overall, this sensitivity analysis confirms that φ=0 is not merely a value consistent with CSO’s own reported parameter settings at this study’s problem dimensionality, but the empirically best-performing choice on both datasets.

## 5. Discussion

### 5.1. On the Comparison Between CSO and the Baselines

CSO’s advantage over the five baselines is dataset-dependent. On Naranjo, it attains the highest mean balanced accuracy of all six methods, 0.847 versus 0.827–0.843 for the baselines (Figure 9); on Oxford it instead ranks second at 0.810, behind HybridGWO’s 0.815 (Figure 5). A paired Wilcoxon signed-rank test on balanced accuracy, matched by seed across algorithms, confirms this asymmetry. On Oxford, CSO significantly outperforms BGWO with p=0.048, QMFO with p=0.007, and BPSO with p=0.036, and is on the margin against BBOA with p=0.050; only its comparison with QMFO survives a Bonferroni-corrected threshold of α=0.01 for the five simultaneous comparisons. Against HybridGWO, CSO is statistically indistinguishable at p=0.51, a near-even 10/20 pairwise split—the only baseline not clearly outperformed on either dataset. On Naranjo, CSO significantly outperforms all five baselines, of which the comparisons against QMFO, BPSO, and BBOA remain significant under the same Bonferroni-corrected threshold.

This pattern is consistent with the higher dimensionality of Naranjo’s encoding: at D=45 acoustic features, versus D=22 for Oxford, the joint search space of Section 3.3 is roughly twice as large, giving CSO’s per-particle searched transfer function, which allows each candidate solution to pick whichever of five binarization rules suits its own region of the space, instead of every particle sharing one fixed rule, with correspondingly more room to specialize. On the smaller Oxford space, this adaptive advantage is thinner, and HybridGWO’s fixed WOA-to-GWO cascade schedule is competitive without it.

CSO’s efficiency profile, demonstrated in Figure 7 and Figure 11, also differs by dataset. On Oxford, it selects the smallest mean feature ratio of all six methods while simultaneously improving accuracy; that is, no accuracy–parsimony trade-off is paid. On Naranjo, it selects more features on average than BBOA or BPSO, trading some parsimony for its accuracy lead. Because the fitness function in Equation (6) weights balanced accuracy nine times as heavily as feature ratio, with ω=0.9, this is the expected behavior, not an inconsistency: whenever a larger feature subset yields a material accuracy gain, the search is designed to keep it. QMFO selects the largest feature subsets on both datasets without a corresponding accuracy advantage, suggesting its search dynamics are comparatively less effective than the other methods at pruning uninformative features under this joint encoding.

A related pattern in Figure 4 and Figure 8 merits a brief note: BPSO and BBOA reach a markedly better mean best fitness within the first few evaluations, then plateau early and are overtaken by BGWO, HybridGWO, and CSO. This follows directly from Hashemi et al.’s [18] hybrid S-/V-shaped transfer function, which binarizes each individual’s position twice per generation, once under each of Equations (2) and (3), and keeps whichever fitness is lower. This best-of-two order statistic cannot score worse than a single draw, but it also costs two fitness evaluations per individual instead of one, so under the shared evaluation budget $FE_{max}$ BPSO/BBOA complete roughly half as many generations as the other methods. Their early lead is therefore a one-time artifact of this selection pressure, not evidence of a more effective search, consistent with their curves flattening earliest while every other method keeps improving.

This comparison holds the classifier-decoding scheme fixed across all six methods: Section 3.3 decodes the last five dimensions identically for every algorithm in this study, as independent on/off switches combined by equal-weight soft voting, so the only axis that differs between the proposed method and the five baselines is the transfer function—each baseline uses the single, fixed transfer function specified in its own source publication, listed in Table 1, while the proposed method instead makes this a per-particle search decision. This is the intended point of contrast, not a confound to be controlled away: Table 2 and Table 3 reflect the choice a practitioner actually faces, between adopting one of five existing, hand-tuned literature pipelines wholesale or adopting the proposed pipeline, which removes transfer-function selection as a manual design decision. As evidence that this per-particle search is doing genuine work instead of converging on one fixed choice, the best-found solution’s transfer function is not constant across the proposed method’s 20 runs on either dataset: on Oxford it is hard-threshold in 12/20 runs, s-shaped-Hashemi in 3, V-shaped-tanh in 2, V-shaped-erf in 2, and s-shaped-classic in the remaining 1; on Naranjo it is hard-threshold in 11/20 runs, s-shaped-Hashemi in 5, V-shaped-tanh in 3, and s-shaped-classic in the remaining 1. No single transfer function dominates every run on either dataset.

Table 6 reports how often each base classifier is selected by the proposed method’s active-classifier record from Section 3.8, across its 20 runs per dataset. The two datasets favor different classifier combinations: KNN and logistic regression dominate on Oxford, selected in 65% and 60% of runs respectively, whereas SVM dominates on Naranjo at 80% of runs, with KNN a secondary contributor at 60%. The mean ensemble size is close to two classifiers on both datasets, 1.95 on Oxford and 1.90 on Naranjo, and the two tree-ensemble methods, random forest and XGBoost, are selected in only a quarter of Oxford’s runs and never selected at all across Naranjo’s 20 runs. This is consistent with this study’s modest subject counts, discussed as a limitation in Section 5.4, not warranting random forest’s/XGBoost’s additional model capacity relative to the simpler margin- and neighbor-based classifiers on these two datasets.

Reference [27] is the study that introduced the Naranjo dataset used throughout Section 4.2; beyond citing it in Section 3.1 as the dataset’s source, we contrast our approach against it methodologically here. Naranjo et al.’s own subject-based Bayesian latent-variable model addresses this dataset’s repeated-measures structure statistically, fitting a model via Gibbs sampling across each subject’s three recordings jointly, instead of through a resampling-based validation design. The proposed method instead addresses this same repeated-measures structure at the evaluation-protocol level, through the subject-grouped 5-fold cross-validation of Section 3.2, adopted specifically to prevent within-subject leakage across folds as discussed further in Section 5.3, while searching the feature subset, classifier-ensemble membership, and transfer function jointly instead of fitting a single fixed classification model. These are two structurally different responses to the same three-recordings-per-subject design: a bespoke statistical model built around it, versus a general wrapper-based search framework whose validation protocol is adapted to respect it.

### 5.2. Cross-Run Feature-Selection Stability and Physiological Plausibility

Section 3.8 noted that this study’s main comparison logs the selected feature *count* for every run but not the identity of *which* features are selected, so which features drive the proposed method’s performance could not previously be assessed across runs, only for a single hand-picked one. To close this gap, the proposed method was re-run for all 20 previously recorded seeds on both datasets, this time with the full feature mask persisted with for every run instead of only the feature count.

Agreement between any two runs’ full feature subsets is weak: the mean pairwise Kuncheva Consistency Index, a chance-corrected measure of subset agreement averaged over all 202=190 run pairs, is 0.138 on Oxford and 0.112 on Naranjo, both far below the range that would indicate two runs tend to select essentially the same subset. Concretely, which secondary features accompany the core signal varies considerably from run to run, so no single run’s complete feature subset, including the single best-performing run examined in an earlier version of this analysis, should be read as *the* interpretable signature of this pipeline.

At the level of individual features, however, a much sharper pattern emerges. If a subset of a dataset’s average selected size were drawn uniformly at random, each feature would be selected in only about 22.5% of runs on Oxford (mean subset size 4.95/22) or 26.1% on Naranjo (mean subset size 11.75/45); a handful of features are selected far above this chance baseline on both datasets. Table 7 lists every feature selected at or above 1.5× its dataset’s chance baseline. Pitch period entropy (PPE), a nonlinear-dynamical measure of aperiodic pitch period variability, tops both datasets: 75% of runs on Oxford (3.3× baseline) and 70% on Naranjo (2.7× baseline, tied with Delta1), consistent across two datasets with different subjects, recording protocols, and feature sets.

This composition is consistent with the physiological picture already established in this study’s related work: nonlinear dynamical measures make up the majority of Oxford’s stable core (PPE, spread1, RPDE, and D2, 4 of 6 features), and PPE tops Naranjo’s core as well, consistent with these measures being more sensitive than jitter/shimmer alone to the aperiodic, incomplete-glottal-closure component of Parkinsonian phonation [9]. Naranjo’s core additionally includes two delta-MFCC features, Delta1 and Delta11, a plausible link to hypokinetic dysarthria’s characteristic rigidity and reduced articulator range of motion [9], since a delta coefficient is precisely a measure of articulatory movement rate; a noise/glottal-closure measure, HNR35; two spectral-envelope coefficients, MFCC5 and MFCC10; and two classical amplitude-perturbation measures, Shim_APQ5 and Shi_APQ11, so the stable core spans multiple feature families instead of concentrating on one.

This reframes what this study can defensibly claim about interpretability. The single best-performing run per dataset examined in an earlier version of this analysis (balanced accuracy 0.858 on Oxford, seed 2476; 0.863 on Naranjo, seed 2776) remains a valid, high-performing solution, but its own feature subset does not include PPE on either dataset, despite PPE being the single most consistently selected feature on both. A single run is therefore a poor basis for an interpretability claim; the frequency-ranked core of Table 7, and PPE’s dominance within it specifically, is the evidence this study now offers for physiologically grounded feature selection, not any one run’s particular subset.

### 5.3. Clinical Implications

Across both datasets, the balanced accuracies attained by every method in this comparison range from roughly 0.79 to 0.85, as summarized in Table 2 and Table 3, consistent with the performance generally reported for voice-based machine learning screening of PD in the wider literature [40]. Because this level of performance is drawn from two small, single-recording-protocol, retrospective public datasets of 31 and 80 subjects each, a limitation discussed further in Section 5.4, we read it as an initial, encouraging level of evidence, not a validated clinical estimate: it is sufficient to support a low-cost, non-invasive triage or monitoring tool, but falls well short of a diagnostic-grade instrument capable of substituting for the MDS Clinical Diagnostic Criteria discussed in Section 2.1. Accordingly, the intended clinical role of a pipeline such as this one is as a first-pass flag that prompts referral for clinical evaluation, or as a between-visit monitoring signal in telemedicine and home-recording settings where repeated in-person motor examination is impractical, pending external validation on a prospectively collected, multi-site cohort, not as a standalone diagnostic decision-maker.

From this clinical perspective, the subject-grouped cross-validation protocol described in Section 3.2 is not a minor implementation detail. Because Oxford’s subjects contribute an average of six recordings each, and Naranjo’s exactly three, a record-level split would allow recordings from the same speaker to appear in both the training and validation folds, allowing the classifier to partly learn speaker identity instead of PD status and thereby inflating every metric in Table 2 and Table 3 relative to the pipeline’s true accuracy on a genuinely new patient. Every result reported here instead estimates accuracy on speakers unseen during that fold’s training—the quantity that actually matters for deployment on a new patient, not on a new recording from a patient already seen.

Finally, the dataset-dependent ranking discussed in Section 5.1—where HybridGWO is competitive on Oxford but not on Naranjo, and CSO’s advantage strengthens with the higher-dimensional Naranjo encoding—cautions against treating any single method, including the one proposed here, as universally best for voice-based PD screening. A practitioner adapting this pipeline to a new population or recording protocol should budget for re-running this kind of multi-method comparison on their own data, instead of assuming that the ranking observed on Oxford and Naranjo will transfer unchanged.

### 5.4. Limitations and Future Work

Although the pipeline proposed in this study achieves strong performance on the PD diagnosis task, it is not without limitations. Foremost among these is external validity: both Oxford and Naranjo are public, retrospective, single-recording-protocol datasets with a modest number of subjects, 31 and 80 respectively, and neither the six-method comparison of Section 4 nor the φ sensitivity analysis of Section 4.3 has been repeated on a prospectively collected cohort, a different recording device or microphone, a different language, or a clinical population with comorbid conditions that could confound acoustic features. This limitation is shared by the broader voice-based PD literature, not specific to this study [40], but it means that the balanced accuracies in Table 2 and Table 3 should be read as evidence that the joint optimization paradigm works on these two datasets, not as an estimate of accuracy on an arbitrary future population.

A further limitation concerns computational cost. The multi-classifier soft-voting ensemble trains up to five base classifiers per fitness evaluation, and every optimizer in this study spends 3000 such evaluations per run. This is a one-time, offline search cost, not a per-patient inference cost, since only the final selected classifier(s) are needed at deployment time; it does mean, however, that reproducing or extending this comparison—for instance to more datasets, more φ values, or more baselines—is markedly more expensive than a feature-selection-only wrapper study would be. This same cost is why a full decoupling analysis of the joint encoding’s three components, e.g., CSO under each fixed transfer function versus the searched transfer function, is not attempted in this revision: at the 20-seeds-per-dataset protocol used throughout this study, each additional component-isolating configuration adds a further 20 runs per dataset at this same 1.7–5.8-h-per-run cost, and we plan to pursue this analysis as a dedicated follow-up study. Finally, this study addresses a single modality, sustained-phonation acoustic features; PD is a multi-system disorder with established non-vocal digital biomarkers as well [15], and combining voice with gait, tremor, or handwriting features within the same joint optimization paradigm introduced here is a natural direction for future work.

## 6. Conclusions

This study proposed a joint transfer function-, feature-, and classifier-ensemble-selection pipeline for voice-based Parkinson’s disease screening, addressing a gap identified in Section 2.3: published metaheuristic pipelines for this task almost universally optimize the feature subset alone, leaving the classifier and the transfer function that binarizes the search fixed by hand. The proposed encoding instead lets a single, plain Competitive Swarm Optimizer search all three jointly, extended with a per-particle transfer-function-selector segment so that each candidate solution can pick whichever of five binarization rules suits its own region of the search space, instead of the entire population sharing one rule fixed before the search begins.

Across 20 independent runs per method on two public, subject-grouped datasets, the proposed method was compared against five baselines from the literature under identical encoding, fitness function, and evaluation-budget protocols. CSO attained the highest mean balanced accuracy on the Naranjo dataset and the second-highest on the Oxford dataset. This dataset-dependent pattern, examined in Section 5.1, is consistent with CSO’s per-particle searched transfer function having more room to specialize as the joint search space grows with the number of acoustic features. A dedicated sensitivity analysis further confirmed that φ=0, the value consistent with the original CSO paper’s own reported parameter settings at this study’s problem dimensionality, is also the empirically best-performing choice for this joint optimization task on both datasets.

These results support the central claim of this work: jointly searching the feature subset, classifier configuration, and transfer function within one encoding is a competitive, and on the higher-dimensional dataset tested here a favorable, alternative to the prevailing practice of optimizing feature selection alone. At the same time, Section 5.4 identifies concrete limitations that temper this claim: both datasets are public, retrospective, and drawn from a single recording protocol each, so external validation on prospectively collected, multi-site, or multi-language cohorts remains necessary before this pipeline could be considered for real screening use. 

## Figures and Tables

**Table 1:** Hyperparameters of algorithms.

Algorithm	Hyperparameters
BGWO	*a*: 2→0
HybridGWO	WOA: b=1.0; GWO: *a*: 2→0
QMFO	$a_{1}=1.0$, $a_{2}=1.5$, β=2.0, dance=5.0,
	fl=1.0, rotation step =0.15
BPSO	w=0.7, $c_{1}=2.5$, $c_{2}=1.6$
BBOA	c=0.01, a∈[0.1,0.3], p=0.6
CSO	φ=0 (as suggested in [38] when D≤100)

**Table 2:** Mean ± standard deviation of balanced accuracy, F1, ROC-AUC, and selected feature ratio on the Oxford dataset (n=20 runs per algorithm). Bold marks the best value per column.

Algorithm	Balanced Accuracy	F1	ROC-AUC	Feature Ratio
BGWO	0.801±0.020 ^†^	0.913±0.009	0.846±0.040	0.227±0.064
HybridGWO	0.815±0.021	0.920±0.010	0.863±0.032	0.255±0.104
QMFO	0.787±0.032 ^‡^	0.909±0.015	0.855±0.035	0.300±0.076
BPSO	0.798±0.029 ^†^	0.912±0.012	0.860±0.038	0.248±0.141
BBOA	0.796±0.023 ^†^	0.910±0.015	0.852±0.040	0.230±0.137
CSO	0.810±0.025	0.918±0.013	0.861±0.037	0.225±0.130

^†^ p<0.05 vs. CSO; ^‡^ p<0.05 vs. CSO and survives a Bonferroni-corrected threshold of α=0.01 for the five simultaneous comparisons, via a paired Wilcoxon signed-rank test on balanced accuracy matched by seed and detailed further in Section 5.1. The unmarked row, HybridGWO, is not significantly different from CSO.

**Table 3:** Mean ± standard deviation of balanced accuracy, F1, ROC-AUC, and selected feature ratio on the Naranjo dataset (n=20 runs per algorithm). Bold marks the best value per column.

Algorithm	Balanced Accuracy	F1	ROC-AUC	Feature Ratio
BGWO	0.840±0.009 ^†^	0.836±0.009	0.864±0.015	0.288±0.052
HybridGWO	0.843±0.007	0.840±0.008	0.861±0.014	0.310±0.059
QMFO	0.833±0.008 ^‡^	0.831±0.008	0.858±0.013	0.377±0.062
BPSO	0.830±0.009 ^‡^	0.828±0.009	0.856±0.015	0.234±0.097
BBOA	0.827±0.008 ^‡^	0.824±0.007	0.859±0.013	0.177±0.051
CSO	0.847±0.012	0.843±0.012	0.862±0.020	0.261±0.066

^†^ p<0.05 vs. CSO; ^‡^ p<0.05 vs. CSO and survives a Bonferroni-corrected threshold of α=0.01 for the five simultaneous comparisons, via a paired Wilcoxon signed-rank test on balanced accuracy matched by seed and detailed further in Section 5.1. The unmarked row, HybridGWO, is not significantly different from CSO.

**Table 4:** Mean ± standard deviation of best fitness, balanced accuracy, and selected feature ratio against φ on the Oxford dataset (n=20 runs per φ value). Bold marks the best value per column.

φ	Best Fitness	Balanced Accuracy	Feature Ratio
0.00	0.194±0.025	0.810±0.025	0.225±0.130
0.05	0.201±0.023	0.797±0.027	0.186±0.079
0.10	0.205±0.028	0.796±0.030	0.216±0.094
0.15	0.203±0.020	0.797±0.023	0.200±0.076
0.20	0.201±0.023	0.802±0.027	0.234±0.088
0.25	0.210±0.026	0.794±0.031	0.250±0.068
0.30	0.209±0.022	0.792±0.024	0.214±0.068
0.35	0.202±0.026	0.803±0.029	0.245±0.116
0.40	0.206±0.024	0.796±0.032	0.218±0.113
0.45	0.206±0.021	0.796±0.023	0.223±0.101
0.50	0.214±0.018	0.784±0.026	0.195±0.093

**Table 5:** Mean ± standard deviation of best fitness, balanced accuracy, and selected feature ratio against φ on the Naranjo dataset (n=20 runs per φ value). Bold marks the best value per column.

φ	Best Fitness	Balanced Accuracy	Feature Ratio
0.00	0.164±0.012	0.847±0.012	0.261±0.066
0.05	0.168±0.013	0.845±0.013	0.283±0.050
0.10	0.168±0.016	0.845±0.013	0.277±0.079
0.15	0.169±0.014	0.843±0.015	0.277±0.078
0.20	0.171±0.014	0.842±0.014	0.284±0.082
0.25	0.172±0.009	0.840±0.013	0.280±0.060
0.30	0.174±0.013	0.839±0.017	0.284±0.085
0.35	0.170±0.011	0.842±0.013	0.278±0.065
0.40	0.173±0.014	0.838±0.017	0.277±0.095
0.45	0.175±0.011	0.839±0.014	0.302±0.061
0.50	0.174±0.015	0.838±0.016	0.279±0.072

**Table 6:** Classifier selection frequency for the proposed method, CSO, across its 20 runs per dataset, Section 5.1, from the active-classifier record of Section 3.8.

Classifier	Oxford (*n* = 20)	Naranjo (*n* = 20)
SVM	4 (20%)	16 (80%)
Random Forest	5 (25%)	0 (0%)
XGBoost	5 (25%)	0 (0%)
KNN	13 (65%)	12 (60%)
Logistic Regression	12 (60%)	10 (50%)
Mean ensemble size	1.95	1.90

**Table 7:** Acoustic features selected at or above 1.5× their dataset’s chance-level baseline selection frequency across the proposed method’s 20 runs per dataset, Section 5.2, grouped by measurement category, with their reported physiological correlate in Parkinsonian dysphonia.

Feature	Freq.	Measurement Category	Physiological Correlate
*Oxford (chance baseline 22.5%; 6 of 22 features at or above 1.5× baseline)*			
PPE	75%	Nonlinear dynamics	Aperiodic, incomplete glottal closure
spread1	45%	Nonlinear dynamics	Aperiodic, incomplete glottal closure
MDVP:Fo(Hz)	40%	Fundamental frequency (mean level)	Altered pitch linked to laryngeal rigidity (monopitch)
Shimmer:DDA	35%	Classical perturbation (amplitude)	Irregular vocal-fold vibration
RPDE	35%	Nonlinear dynamics	Aperiodic, incomplete glottal closure
D2	35%	Nonlinear dynamics	Aperiodic, incomplete glottal closure
*Naranjo (chance baseline 26.1%; 8 of 45 features at or above 1.5× baseline)*			
PPE	70%	Nonlinear dynamics	Aperiodic, incomplete glottal closure
Delta1	70%	Delta-MFCC (rate of change)	Slowed or reduced articulatory movement rate
Delta11	65%	Delta-MFCC (rate of change)	Slowed or reduced articulatory movement rate
HNR35	60%	Noise/glottal-closure	Aperiodic, incomplete glottal closure
MFCC5	55%	Spectral envelope	Articulatory configuration (vocal-tract shape)
MFCC10	50%	Spectral envelope	Articulatory configuration (vocal-tract shape)
Shim_APQ5	40%	Classical perturbation (amplitude)	Irregular vocal-fold vibration
Shi_APQ11	40%	Classical perturbation (amplitude)	Irregular vocal-fold vibration

## Data Availability

The datasets analyzed in this study are publicly available in the UCI Machine Learning Repository: the Oxford Parkinson’s Disease Voice Dataset at https://archive.ics.uci.edu/dataset/174/parkinsons (accessed on 9 August 2026), and the Naranjo Replicated Acoustic Features Dataset at https://archive.ics.uci.edu/dataset/489/parkinson+dataset+with+replicated+acoustic+features (accessed on 9 August 2026). The code and experimental results are openly available in Zenodo at https://zenodo.org/records/21897611 (accessed on 9 August 2026).
